# Supplementary material for: Experiences and perspectives on chimeric antigen receptor (CAR) T-cell therapy among recipients, carers and referrers (RE-TELL): a qualitative study to inform CAR T-cell service design
Source: BMJ Open. 2024 Jan 23;14(1):e071112. doi: 10.1136/bmjopen-2022-071112 (PMC10824048; doi:10.1136/bmjopen-2022-071112)
Supplement: Supplementary data [file bmjopen-2022-071112supp001.pdf]

**Supplementary file 1:** Semistructured interview guide

**For patients and support persons**

**Introduction and explanation of session**
**Entry questions**

- Age
- Ethnicity
- Education
- Whether received treatment or supported someone through treatment

**Experiences through treatment**

- Describe your CAR T-cell treatment
    - When did you first hear about CAR T-cells?
    - How did you travel to obtain CAR T-cells?
    - How did you find the treatment process?
        - How long did you spend in another city obtaining CAR T-cell treatment?
    - How did you find returning to your home city after treatment?
        - How well did your different health professionals co-ordinate together?
        - How did you find the follow-up period after having treatment with CAR T-cells?
- What was the emotional impact of the treatment?
    - Were your expectations met by the treatment?
    - Is there anything that could have made the treatment easier for you?
- What was the impact of your treatment on your support person and wider whānau/family?
- What was the financial impact of your treatment?
    - What support was provided during this time?
    - What other supports could have helped?

**Treatment aspects that worked well**

- What parts of your treatment journey do you think worked well?
- What parts of your treatment should be preserved, or kept, if more people were treated with CAR T-cells in the future?

**Treatment aspects that could be improved upon**

- What parts of your treatment do you think could be improved upon?

- How could the process of travelling to-and-from the CAR T-cell treating centre be improved?
- What parts of your medical treatment did you find most challenging?
- Were the explanations about CAR T-cells accurate for your experience?
    - If not, why not?

    - What else would you have wanted to know about before receiving CAR T-cell therapy?
- Were there any emotional supports that could have made this process easier for you?
- What challenges did you face in accessing CAR T-cells that you think could be avoided in future?
- Did you feel your culture was respected throughout your treatment?
    - If not, why not?
- Did you feel your religion was respected throughout your treatment?
    - If not, why not?
- What challenges did you face in accessing CAR T-cell treatment?
    - How could this be addressed in future?

**For clinicians and administrators**

**Introduction and explanation of session**
**Entry questions**

- Age
- Ethnicity
- Education
- Role in CAR T-cell treatment
- Experience with CAR T-cell treatment

**System flow**

- How well does the current healthcare system facilitate access to CAR T-cell therapy?
- How efficient do you think the current system is?
- What are the current barriers to patients accessing CAR T-cell treatment?
- What are the current barriers to patients moving through CAR T-cell treatment?
- What factors help patients access CAR T-cell treatment?
- Do you think current access to CAR T-cell therapy is equitable?
    - If not, what are the main current barriers?
        - How could these be addressed?

**Staff experience of the CAR T-cell therapy pathway**

- Did you have all the information you needed to help your patient access CAR T-cell treatment?
    - What else would you have wanted to know prior to the treatment?
- What resources could help improve the CAR T-cell treatment pathway from a practical perspective?
- What was your experience of the CAR T-cell treatment programme from an emotional perspective?
    - Are support resources needed for staff during this treatment?
