# Supplementary material for: Experiences and perspectives on chimeric antigen receptor (CAR) T-cell therapy among recipients, carers and referrers (RE-TELL): a qualitative study to inform CAR T-cell service design
Source: BMJ Open. 2024 Jan 23;14(1):e071112. doi: 10.1136/bmjopen-2022-071112 (PMC10824048; doi:10.1136/bmjopen-2022-071112)
Supplement: Author's manuscript [file bmjopen-2022-071112.draft_revisions.pdf]

BMJ Open is committed to open peer review. As part of this commitment we make the peer review history of every article we publish publicly available.

When an article is published we post the peer reviewers' comments and the authors' responses online. We also post the versions of the paper that were used during peer review. These are the versions that the peer review comments apply to.

The versions of the paper that follow are the versions that were submitted during the peer review process. They are not the versions of record or the final published versions. They should not be cited or distributed as the published version of this manuscript.

BMJ Open is an open access journal and the full, final, typeset and author-corrected version of record of the manuscript is available on our site with no access controls, subscription charges or pay-per-view fees (http://bmjopen.bmj.com).

If you have any questions on BMJ Open's open peer review process please email info.bmjopen@bmj.com

BMJ Open

A qualitative study of chimeric antigen receptor (CAR) T-cell recipients, carers and referrers (RE-TELL): Implications for CAR T-cell service design

Journal:	*BMJ Open*
Manuscript ID	bmjopen-2022-071112
Article Type:	Original research
Date Submitted by the Author:	12-Jan-2023

Complete List of Authors:	Fyfe, Robert; Malaghan Institute of Medical Research; Victoria University of Wellington<br>Anstis, Olivia; Deloitte<br>Kapadia, Kushant; Deloitte<br>Jordan, Mallory; Deloitte<br>Sword, Danielle; Malaghan Institute of Medical Research<br>Weinkove, Robert; Malaghan Institute of Medical Research; Te Whatu Ora Health New Zealand Capital Coast and Hutt Valley, Te Rerenga Ora Blood & Cancer Centre

Keywords:	QUALITATIVE RESEARCH, HAEMATOLOGY, Organisation of health services < HEALTH SERVICES ADMINISTRATION & MANAGEMENT, Health Equity, Lymphoma < HAEMATOLOGY

SCHOLARONE™
Manuscripts

I, the Submitting Author has the right to grant and does grant on behalf of all authors of the Work (as defined in the below author licence), an exclusive licence and/or a non-exclusive licence for contributions from authors who are: i) UK Crown employees; ii) where BMJ has agreed a CC-BY licence shall apply, and/or iii) in accordance with the terms applicable for US Federal Government officers or employees acting as part of their official duties; on a worldwide, perpetual, irrevocable, royalty-free basis to BMJ Publishing Group Ltd ("BMJ") its licensees and where the relevant Journal is co-owned by BMJ to the co-owners of the Journal, to publish the Work in this journal and any other BMJ products and to exploit all rights, as set out in our licence.

The Submitting Author accepts and understands that any supply made under these terms is made by BMJ to the Submitting Author unless you are acting as an employee on behalf of your employer or a postgraduate student of an affiliated institution which is paying any applicable article publishing charge ("APC") for Open Access articles. Where the Submitting Author wishes to make the Work available on an Open Access basis (and intends to pay the relevant APC), the terms of reuse of such Open Access shall be governed by a Creative Commons licence – details of these licences and which Creative Commons licence will apply to this Work are set out in our licence referred to above.

Other than as permitted in any relevant BMJ Author's Self Archiving Policies, I confirm this Work has not been accepted for publication elsewhere, is not being considered for publication elsewhere and does not duplicate material already published. I confirm all authors consent to publication of this Work and authorise the granting of this licence.

For peer review only

TITLE

A qualitative study of chimeric antigen receptor (CAR) T-cell recipients, carers and referrers (RE-TELL): Implications for CAR T-cell service design

Authors

Fyfe R[1,2,3], Anstis O[4], Kapadia K[4], Jordan M[4], Sword D[1], Weinkove R[1,2,5].

Affiliations

1. Malaghan Institute of Medical Research, Wellington, New Zealand

2. Te Rerenga Ora Blood & Cancer Centre, Te Whatu Ora Health New Zealand – Capital, Coast and Hutt Valley, Wellington, New Zealand

3. Faculty of Science, Victoria University of Wellington, Wellington, New Zealand

4. Deloitte, Auckland, New Zealand.

5. Department of Pathology and Molecular Medicine, University of Otago Wellington, Wellington, New Zealand

Corresponding author

Dr Robert Fyfe

Email: Robert.Fyfe@ccdhb.org.nz

Telephone: +64 4 385 5999

Address: Te Rerenga Ora Blood & Cancer Centre, Te Whatu Ora Health New Zealand – Capital, Coast and Hutt Valley, 49 Riddiford Street, Wellington, New Zealand

Word, figure and reference counts

Abstract: 292 words

Article: 4820 words

Tables: 1

References: 21

For peer review only

Supplemental files: 3

For peer review only

ABSTRACT

Objectives:

To understand patient, support person, clinician and co-ordinator experiences and perspectives of chimeric antigen receptor (CAR) T-cell therapy, to inform design of a clinical CAR T-cell service in Aotearoa New Zealand.

Methods:

Qualitative interviews were undertaken with 19 New Zealand participants comprising: five CAR T-cell recipients, three support persons, six clinicians and five co-ordinators (including transplant co-ordinator, travel assistance, and charitable organisations). 4 participants identified as Māori. Semi-structured interviews focussed on themes of: experience through treatment; elements that work well and those that could be improved upon. Interviews were analysed using thematic analysis to identify key themes. A workshop was held with participants to gain reflections on the interim analysis and proposed improvements.

Results:

Thematic analysis identified three global themes. The first, Accessing CAR T-cell therapy, identified potential sources of inequity including geographic, financial and informed consent barriers. The second, Current experiences with CAR T-cell therapy, identified an easier treatment experience compared to alternatives; an underwhelming cell administration process; frustration with inpatient monitoring; burden on support persons; and importance of "bridge" organisations such as charities and patient support groups. The third, Future improvements, suggested: improved geographical access to CAR T-cell therapy, while retaining consolidated clinician experience; a

'dashboard' with key information on CAR T-cell treatment, timeframes and manufacture; a health navigator to co-ordinate non-medical aspects of treatment and signpost care; embedding of indigenous data sovereignty and ownership of cells; a ceremony for cell infusion, incorporating family involvement and Māori cultural elements; and outpatient administration and monitoring where possible.

Conclusion:

This study has documented the current experience of New Zealanders receiving CAR T-cell therapy, and identified opportunities for future service development. These insights are relevant to service design both within Aotearoa New Zealand, and for other countries developing equitable CAR T-cell services.

Key words

Receptors, Chimeric Antigen; Immunotherapy, Adoptive; Qualitative Research; Technology, high cost; Genetic Therapy; Health Inequities; Health Services, Indigenous.

For peer review only

STRENGTHS AND LIMITATIONS OF THIS STUDY

- Strengths
  - Diverse viewpoints pertaining to CAR T-cell therapy in Aotearoa New Zealand
  - Many facets of treatment experience explored
  - Specific focus on strategies to improve indigenous Māori health outcomes
- Limitations
  - Data saturation may not have been reached for all areas
  - Further research required to develop more solutions to existing challenges

For peer review only

INTRODUCTION

Chimeric antigen receptor (CAR) T-cell therapy has become a standard of care for some relapsed and refractory B-cell malignancies [1,2]. As a personalised cell and gene therapy involving gene modification of a patient's own T-cells, access to treatment is limited by logistical challenges and cost, while varied concerns regarding the technology need to be addressed, including equity of access, optimising the patient and support person experience, and cell and data sovereignty [3,4,5]. Internationally, CAR T-cell therapy provision has not consistently ensured equity of access [6,7], and equity has been highlighted as a key area of focus for ongoing development of this treatment modality [8].

Aotearoa New Zealand does not yet have a national service for standard of care CAR T-cell delivery, with access limited to clinical trial use [9] or overseas treatment. Within Aotearoa New Zealand, cancer-specific mortality is higher for indigenous Māori than for non-Māori, including for lymphoma and myeloma [10]. As a result, there is a strong focus on designing healthcare services to rectify this inequity [11]. A future national CAR T-cell service should be intentionally designed to ensure the service results in health gains for Māori, and does not exacerbate health inequities.

While the challenges of delivering equitable access to CAR T-cell therapy have been considered worldwide [12,13], the Aotearoa New Zealand healthcare system has unique characteristics, such as: an imperative to promote indigenous health due to the government's obligations to Te Tiriti o Waitangi/the Treaty of Waitangi (the nation's founding document, which guarantees Māori the right to access to equitable healthcare and health outcomes); low public pharmaceutical funding compared to some high income countries [14]; and wide geographic dispersal [15].

For peer review only

We aimed to assess the current experiences of CAR T-cell therapy for patients, support persons, clinicians and administrators within Aotearoa New Zealand, to develop insights into treatment experience, needs, and barriers to treatment; and to identify potential improvements for future services. Our overall goal was to inform design of a future national CAR T-cell service. We conducted semi-structured qualitative interviews, assessed these interviews with thematic analysis, and further refined this analysis through a co-design workshop with participants, where the interim results of the thematic analysis were presented to participants for comment.

METHODS

Study design

We intended to recruit a total of 19 participants, including 5 patients, 3 support persons, 6 clinicians and 5 administrators, to provide a diversity of perspectives on CAR T-cell therapy. Participants were selected using purposive sampling to encompass a range of experiences (treated in Aotearoa New Zealand or overseas; different outcomes after treatment; different roles in a patient's care) and a diversity of backgrounds (age; ethnicity; gender; urban and rural).

A co-design methodology was chosen to take a partnership approach with patients and support persons, to understand patient experience, and to use this to develop potential improvements. Following the methodology of Boyd et al [16], the project followed the six main elements of: Engage, Plan, Explore, Develop, Decide, Change. Engage refers to establishing relationships with patients and staff to improve health services. Plan involves generating ideas about the goals of improvement work and strategies to

For peer review only

achieve it. Explore refers to understanding patient and staff experiences, to identify areas for improvement. Develop involves turning ideas into specific improvements. Decide involves a choice between improvements and how to implement them. Change means then turning ideas into action.

Data collection

Utilising semi-structured interviewing techniques, we interviewed participants following the themes of: treatment experience; elements that worked well; elements that could be improved on; practical needs; informational needs; emotional experiences; cultural elements; and system flow. The full interview guide is provided as Supplemental Appendix 1. Interview data were analysed using thematic analysis, with transcripts themed following the framework provided by Braun and Clarke [17]. All interviews and the workshop were facilitated by a Health Psychologist (OA) registered with the New Zealand Psychologist's Board. Interviews were performed between 13 January 2022 and 8 April 2022. Interviews were conducted via videoconference, as COVID-19 pandemic-related restrictions precluded in-person interviews. Interviews were audio-recorded, and lasted between 28 and 60 minutes. Interviewees were given the option to review manuscripts and provide comment.

An interim thematic analysis was presented to participants (n = 7) who self-selected to attend a videoconference workshop, in order to gain reflections on the responses and potential service improvements. During this workshop, a graphical representation of the patient journey through CAR T-cell therapy was displayed, as described by RE-TELL participants (Supplementary appendix 2). This served to summarise information gathered during the interviews, to provide logical structure to the themes by mapping them across the treatment timeline, and to help overcome health literacy

For peer review only

barriers. The responses from workshop participants assisted with the formulation of "Table 1, Global Theme 3: Future improvements to CAR T-cell pathway" and the improvements emphasised in the discussion; no new themes were identified during the workshop. The primary themes from this workshop are presented in Results – Co-Design Workshop.

Statistical and ethical considerations

Interviews were transcribed verbatim and analysed using data analysis software NVivo V.11. Transcripts were analysed and coded independently into key themes by RF and OA according to existing guidelines [17, 18]. OA had no prior involvement with the participants or CAR T-cell therapy; RF was the clinical fellow for the ENABLE trial during the study, with clinical involvement with 2 patients and 1 participant. The researchers then met to seek alignment and generate global and sub-themes collaboratively. Subthemes were allocated to provide contextual explanation of key themes, or describe less common yet important topics or emotional descriptors. Themes were formulated into a table including sample belief statements and sample quotes.

Ethical approval for this study was provided by Victoria University of Wellington Human Ethics Committee (ref: 29982) before any study activities. All participants gave written informed consent. The original study protocol is available as supplemental file 3.

Patient involvement

Patients were not involved in setting the research question or formation of the study protocol, but their responses directed the interviews, and provided the themes. We

For peer review only

intend to disseminate the results to study participants in a newsletter suitable for a non-specialist audience.

RESULTS - INTERVIEWS

Baseline characteristics

We enrolled 19 participants, including 5 patients, 3 support persons, 6 clinicians and 5 administrators. 4 participants were Māori, 11 New Zealand European, 2 Asian and 1 other; 12 of 19 participants (63%) were female, 7 male. The highest level of education was secondary education for 4 participants, and tertiary education for 15.

Among the 5 patients interviewed, all had B-cell non-Hodgkin lymphoma and had received CAR T-cell therapy. Their median age was 48 years (range 23-59). Three had received investigational third generation CAR T-cells within the phase 1 'ENABLE' trial in Aotearoa New Zealand [9]; one had received standard of care axicabtagene ciloleucel (axi-cel) in Australia via a government funding mechanism; and one had received lisocabtagene autoleucel in the U.S.A within a clinical trial. Four of the five patients had a complete response to CAR T-cell therapy at the time of interview.

Of the 3 support persons, one had supported a patient through treatment via the ENABLE trial; one through treatment in Australia; and one through treatment in the U.S.A.

The clinicians interviewed comprised four clinical haematologists who had referred patients for CAR T-cell therapy, one palliative care nurse practitioner and one research

For peer review only

nurse. The administrators comprised a charitable services co-ordinator, a bone marrow transplant co-ordinator, a transport co-ordinator and two social workers.

Data saturation

Data saturation occurs when no additional themes emerge from the data. This was not reached in this study for all areas of analysis, and reasons for this are explored in the Discussion. Specifically, many of the subthemes mentioned in Global Theme 3 did not reach saturation, as there were a multitude of potential areas improvements raised, which were not discussed with all participants.

Key domains and themes:

Themes arising from the interviews were organized into three global themes, before subclassification into 8 domains and 23 subthemes (see Table 1). The first global theme, Accessing CAR T-cell therapy, relates to both the challenges that patients and clinicians face in attempting to access CAR T-cell therapy, and the barriers and inequities that may prevent others from accessing the treatment. The second global theme, Current experiences with CAR T-cell therapy, describes the experience of treatment within New Zealand or overseas, including the ancillary services in place to support patients. The final global theme, Future improvements to CAR T-cell therapy, explores the ways that a future national CAR T-cell therapy service might improve treatment delivery.

Table 1: Global themes, key domains, subthemes and sample quotes

Relevant domains	Subtheme	Sample quotes
Global theme 1: Accessing CAR T-cell therapy		
Equity	Potential inequity stemming from lack of access	"To be equitable, you need to make sure it is accessible to everyone, and we can't just rely on clinical trials and compassionate access programmes" [C1].

For peer review only

	Geographic barriers	"The first barrier is distance" [C1]<br>"I understand you can't have CAR T in all centres. I just think one is probably not enough for many patients" [C1]<br>"You are asking someone who has a limited support network to fly… it's hard for the patient psychologically"[C1]
	Informed consent	"As soon as you start talking about how risky things are – a Māori worldview is often quality over quantity…. In a medicolegal worldview, we go into the nth degree about every small little potential risk, such that [Māori] will start to get scared and go well, actually, I don't want this." [C5]<br>"You should give me information that I can just hand to my mother and she understands it" [P1]
	Financial barriers	"People just don't have enough money… for financial support, transitioning from being a full time employee to having to stop." [A4]<br>"We say to patients we have this intensive treatment – it's fully funded, all public… but we have no mechanism to help you stay down here, and you've got to a charity [for accommodation]" [A2]
	Awareness of trial	"At least a couple of patients initiated the conversation about wanting to be part of the CAR T-cell trial" [C4]<br>"People don't know where to look sometimes" [C6]
	Requirement for advocacy	"Our health system requires you to navigate it yourself, it's so patient unfriendly" [P1]
**Global theme 2: Current experiences with CAR T-cell therapy for New Zealanders**		
Patient experience	Underwhelming administration of cells	"It's just a shot in your arm and then you're done… anti-climactic". [P5]
	Treatment experience easier compared to chemotherapy	"It was quite chill… chemo's a hell of a lot worse" [P5]<br>"A walk in the park" [P2]<br>"It was actually the first time through the whole cancer journey that I got to sleep a lot… I just rested" [P3]
	Frustration with prolonged inpatient monitoring	"I was so bored I went out to strip the bed, got some new sheets and made my bed up" [P2]
Support person experience	Emotional burden	"The relief to get him to the hospital and know that somebody else was looking after him, and it wasn't me that was having to keep things going every day" [S3]<br>"There's no care for the carers" [S1]
	Requirement to provide care for patients	"I felt like I'd gone from being a wife to a carer" [S3]
	Role in advocacy for patients	"Half the time I don't think [the patient] knew what day it was" [S3]
Bridges	Charities	"Sometimes patients and families do like to talk to an organization that understands, but is separate to the hospital… so they won't be judged" [A1]<br>"There is a period of time there where the hospital finishes their treating, and they may not be so unwell that a hospice is

For peer review only

		involved, but they're in that middle zone and it's really stressful." [A1]
	National Travel Assistance programme	"It's not meant to cover [all travel costs], but it's meant to … eliminate the barrier of those upfront costs" [A5]
	Patient support groups	"Show respect for what they've gone through and being able to articulate that experience to others" [A1]
Global theme 3: Future improvements to CAR T-cell pathway		
Centralised point of information	Accessible prepared resources	"It's good to have it delivered through a video. It's a bit easier to comprehend" [P1]<br>"Knowledge is power… if you've got the information and know what to expect, it takes a lot of the fear out of the process" [P3]
	Health navigator	"Someone who's thinking about the whole patient's life and experience going through the process, not just the medical side of it… a generalist, and then the specialists are doing their work". [P1]
Alterations to treatment delivery	Outpatient therapy	"I could have done a lot of that as an outpatient" [P2]
	Ceremony for cell infusion	Having family members, and potentially referring clinicians present for cell infusion: "It will show [patients] how important it is" [C5]
	Need for appropriate accommodation	"Really inappropriate [to require family members to sleep on the floor]" [P3]
Service design	Clinical staff experience	"You need to have [dedicated] staff because it's a unique therapy… [bone marrow transplant co-ordinators] couldn't just absorb [a CAR T-cell therapy role], they'd need extra resource". [A2]
Indigenous rights	Data sovereignty	"Is it information for information's sake, or is there a purpose at the end?" [C5]
	Cell sovereignty	"They told us what they were going to do with the stem cells if they didn't need them.. whereas in Australia? We never got those options". [S2]<br>"You almost want a courier tracking system [for cells]" [C5]

<u>Global theme 1: Accessing CAR T-cell therapy</u>

Potential inequity stemming from lack of access: Current mechanisms of access to CAR T-cell therapy in Aotearoa New Zealand are via clinical trial, self-funded treatment overseas, or government funded treatment overseas. There was broad agreement among those interviewed that current access to CAR T-cell therapy is inequitable, with the impression that those able to access treatment are likely to be more health literate, have more financial resources, are more likely to reside in an

For peer review only

urban centre, and exist within societal structures that enable their health literacy and ability to navigate the health system. Without mitigation strategies, this could present greater access barriers to Māori and Pacific peoples.

Requirement for self-advocacy: Noting the barriers to accessing CAR T-cell therapy identified above, a frequently raised theme was that patients and support persons were required to advocate for themselves, either to access CAR T-cell therapy in the first instance, or to shape their clinical care to fit their needs. Although referrals for CAR T-cell therapy occurred via a patient's haematologist/oncologist, several patients had to raise the option of CAR T-cell therapy with their treating clinician before this was considered. Many felt that the in the absence of standard-of-care CAR T-cell therapy in Aotearoa New Zealand, knowledge of referral pathways relies on 'word-of-mouth' communication.

Geographic barriers: Aotearoa New Zealand is a country with a low population density, long transport times between centres, and is distant from international centres that offer CAR T-cell therapies. Geographic inequity was identified as a factor impacting patients' ability to access therapy. Geographic barriers impacted people's finances and ability to care for their children, and resulted in time away from support networks and whānau (family), and concerns about housing and job security.

*Informed consent***:** It was noted that many aspects of accessing treatments in Aotearoa New Zealand require an ability to navigate a complex medical system. In particular, the current process of consent for investigational treatments requires extensive cataloguing of possible risks of therapy. Participants commented that if this is

For peer review only

provided without an equivalent explanation of potential benefits, patients may leave with the understanding that CAR T-cell therapies are riskier than they truly are (rather than accepting that the Western medicolegal paradigm requires robust documentation of each risk) [see: Table 1; global theme 2]. While this issue with informed consent was noted by both NZ European and Māori patients, it was thought that it may have greater impact on Māori patients. Interviewees noted that this could be further exacerbated by differences in health literacy and an inability for many clinicians to provide information within a Te Ao Māori (Māori world) framework, which could contribute to an erosion of trust in the health system. Without mitigation, this could impede Māori access to innovative medical treatments such as CAR T-cell therapy.

Financial barriers: Although patients attempting to access CAR T via clinical trial in Aotearoa New Zealand experienced barriers, these were even more formidable for patients attempting to self-fund CAR T-cell therapy overseas. Among those accessing overseas treatment, patients and family were required to identify overseas hospitals with actively enrolling CAR T-cell therapy programmes; liaise with these hospitals and arrange transfer of medical information; fundraise high sums through combinations of personal funds, fundraising events or crowdfunding; and co-ordinate logistics to arrive at the treating hospital – all while unwell with their disease. Unsurprisingly, it was noted that many who considered exploring treatment via this route were eventually unable to access this.

<u>Global theme 2: Current experiences with CAR T-cell therapy</u>

Treatment experience easier than chemotherapy: The predominant theme regarding the treatment experience was the relative tolerability of CAR T-cell therapy. New

For peer review only

Zealanders who accessed CAR T-cells within the ENABLE trial did so as a last-line therapy, so had experience of numerous chemotherapy cycles, and often of stem cell transplantion. Participants reported that, although there were adverse effects, CAR T-cell therapy was much more comfortable and tolerable than their prior treatments. Indeed, the primary difficulty with CAR T-cell therapy was reported to be the prolonged inpatient monitoring (typically 14 days), or (for the recipient of axi-cel in Australia) monitoring in an Intensive Care Unit for cytokine release syndrome (CRS), while feeling well. Additionally, many of the treatment challenges highlighted were not primarily attributable to physical adverse events, but rather to socioeconomic factors. For those treated in Aotearoa New Zealand, these included: accessing suitable accommodation for patient and support person; affording groceries; and compensating for loss of income while a support person relocated to accompany the patient. For those treated outside Aotearoa New Zealand, these included: identifying overseas treating centres and securing a treatment place; complex and expensive logistical planning; and balancing having a support person at the treating centre to support the patient with maintaining family life at home. It was commented that conducting follow-up in an outpatient setting would have overcome many of the challenges of treatment (see Global Theme 3).

Underwhelming administration of cells: It was noted that the infusion of CAR T-cells could be anti-climactic, since it involved a very brief cell infusion. While some patients found the moment of CAR T-cell administration momentous and significant, others felt it was underwhelming, and detracted from the treatment experience.

For peer review only

Support person experience: Our interviews identified the crucial role that support persons played in the care of patients receiving CAR T-cells. This included assistance with monitoring for CAR T-cell related adverse events, and co-ordinating the many non-medical challenges that arise as a patient goes through cancer treatment. In addition, support persons often played an active role by advocating for the patient. Reckoning with these new responsibilities, while managing the psychological stress of a close family member's life-threatening illness, presented a significant emotional burden for support persons.

Bridges: A characteristic of CAR T-cell therapy treatment for New Zealanders was the need for external organisations to serve as "bridges" to cover gaps in patient care that currently exist in the funded health system. These "bridges" include charitable organisations, such as Leukaemia and Blood Cancer NZ, who provide financial assistance for travel and accommodation costs during treatment, psychosocial support, and participation in support groups for patients to meet other patients receiving similar treatment. Such support groups also arise organically, such as via social media. Community hospices also provide a valuable service providing both end-of-life care and community outreach palliative care input.

The current healthcare system provides a National Travel Assistance programme, which covers a portion of the logistical and accommodation costs for patients and support persons travelling out-of-region for medical treatment. However, the gaps in coverage of this programme result in costs falling to either patients or to charitable organisations.

For peer review only

Global theme 3: Future improvements to CAR T-cell therapy

The final major theme related to improvement opportunities for a future national CAR T-cell therapy service.

Centralised point of information: It was recognized that the introduction of a CAR T-cell therapy should be accompanied by education and support structures to help clinicians and patients unfamiliar with the therapy to access it. For clinicians, this would require visible and accessible specialists to liaise with, and clear and accessible guidance of the referral process. For patients, this involves quality education materials to explain the treatment in understandable terms, preferably in video form in addition to text.

Health navigator: It was identified that a health navigator (sometimes referred to internationally as a "concierge") could serve a role as a patient liaison throughout treatment, coordinating both the medical and non-medical aspects of care (including logistic, financial and psychosocial considerations), providing patient advocacy, and education about aspects of treatment. An idiosyncratic element of CAR T-cell therapy is the cell manufacturing period, leading to a period of uncertainty for patients and referring clinicians as the outcome of CAR T-cell manufacture is awaited. Both clinicians and patients identified a potential role for a 'cell tracker', with which the progress of cell manufacture could be tracked over time, to manage expectations and provide reassurance.

Changes to treatment delivery: Several potential alterations to current CAR T-cell therapy clinical delivery were identified. First was the possibility of conducting a

greater proportion (or even the entirety) of monitoring as an outpatient following cell infusion. This was felt to offer benefits for patient wellbeing as well as for health resourcing. Secondly, to address the underwhelming nature of cell administration, a ceremony for CAR T-cell administration was suggested, to imbue the procedure with a reverence befitting the occasion. This could involve family, friends or referring clinicians, either in person or via videoconference, as well as additional cultural or religious elements such as presence of kaumatua (Māori elders) and a karakia (blessing).

Dedicated clinical staff: It was identified that a CAR T-cell therapy service requires dedicated resourcing and training, rather than unfunded incorporation into overstretched bone marrow transplantation services. Treatment hubs should have dedicated CAR T-cell staff to allow a consolidation of skills and experience, and to ensure safe treatment delivery.

Indigenous sovereignty: Finally, it was identified that a future CAR T-cell service should include provisions to respect indigenous sovereignty over data and cells. For data, this should include anonymizing or de-identification of data shared beyond treating clinicians (unless explicit consent is given), as well as the understanding that data would lead to improvements for the patient group that the data is sourced from. For cells, this would involve explanation of where cells would be located, what processes would be undertaken with them, and what would happen to remaining cells that are not required for their cellular therapy. As an example, one Māori participant underwent a cell collection procedure in Aotearoa New Zealand, before undergoing cell collection for a commercial CAR T-cell therapy overseas. They highlighted that

For peer review only

in Aotearoa New Zealand they were informed about location of their cells during manufacture, and were given the option to either donate or dispose of cells that were not required for treatment. Conversely, during their treatment overseas they were not informed of the location of their cells or the manufacturing progress, nor given options about fate of their cells.

For peer review only

RESULTS – CO-DESIGN WORKSHOP

Participants noted that, for patients and support persons attempting to access CAR T-cell therapy, many of the challenges to be navigated are at or before the start of the pathway, and suggested refinements to the pre-referral process, and monitoring of referrals to ensure access is equitable.

Participants commented that, although many elements of the current pathway could be improved upon, including consent processes, support services and cultural elements, the primary focus of patients and support persons is to access CAR T-cell therapy, and that the need to improve these areas should not prevent initial adoption of the therapy.

Participants were asked to comment on which of the potential improvements (broadly identified in Global Theme 3) they would prioritise. This resulted in strong support for the health navigator role (to assist patients and families with non-medical and medical navigation of the health service and the effect that cancer treatment would have on their lives); the need for culturally appropriate care for all patients; and a centralised dashboard for information (to provide information about how to access CAR T-cell therapy and what treatment would entail). Elements such as a tracker for cells after extraction, and a ceremony around cell infusion, were considered improvements but less crucial to the success of a CAR T-cell therapy programme.

DISCUSSION

Aotearoa New Zealand has an unmet need for funded CAR T-cell therapies for B-cell non-Hodgkin lymphomas and other B-cell malignancies. As we expect CAR T-cell

For peer review only

therapy to become a standard of care in the future, it is important that consideration is given to the perspectives of patients, support persons, clinicians and administrators, in design of a national service. Therefore, we performed this qualitative study to generate insights into the current experiences of CAR T-cell therapy, and the areas which could be improved upon.

Elements of the current service were praised by participants. First, CAR T-cell therapy was considered more tolerable than other treatments such as chemotherapy and autologous stem cell transplantation. Second, the role of charities and other organisations in expanding on the support provided by healthcare providers, and mitigating the challenges facing patients and support persons through treatment was applauded. Third, the use of strategies to respect tissue sovereignty within the ENABLE trial was thought to lay important groundwork for further research in this area in future.

We identified several areas for potential improvement of CAR T-cell delivery. First were strategies to improve communication with patients and referring clinicians, and therefore minimise some of the barriers to access identified in Table 1. Some patients had to ask to be referred for the CAR T-cell clinical trial, suggesting that a more robust system of informing clinicians of open clinical trials is required. For a standard of care service, strategies could include a centralised 'dashboard' for new CAR T referrals (with clear guidance for clinicians on eligibility, process and timeframes), and high-quality educational materials for patients and family in understandable language. Communication could also be improved with a 'tracker' of cell collection and manufacture, which would provide patients and referring clinicians with an insight into timeframes.

For peer review only

Other improvements to improve the treatment experience included a transition to outpatient management, which would result in benefits for patients (improved psychosocial wellbeing and avoidance of infection while in hospital), alongside resourcing benefits (reduced inpatient burden, and less overall staff input). Such a transition would require more robust assistance from a National Travel Assistance programme, to ensure that treatment remains a realistic option for patients with fewer resources. A ceremony for CAR T-cell administration, including involvement of family and referring clinicians, and incorporating cultural or religious elements, may be appreciated by many patients.

Given the ethnic health disparities in Aotearoa New Zealand, including poorer cancer-specific survival for non-Hodgkin lymphoma and myeloma among Māori [10], a national CAR T-cell service will need to be designed with an explicit equity focus to ensure it improves Māori health outcomes. Given the socioeconomic disparities since colonisation, this may require increased support to access treatment and to mitigate financial consequences for the patient and support persons relocating for treatment. It would also require culturally appropriate care within the CAR T-cell therapy team. Patient autonomy over data and cells was strongly valued, with the option to decide on how both are used.

Finally, we identified the need for treating hubs with consolidated experience and skills, to ensure that clinicians can deliver this new therapy safely. However, it was felt that keeping CAR T-cell therapy in a single treating centre, even in a country with a relatively small population such as Aotearoa New Zealand, could perpetuate existing

For peer review only

geographic inequities. Therefore, consideration will need to be given in the medium term to balancing the relative trade-offs between efficiencies gained from consolidated care and the benefits to patients of care closer to home.

One of the key strengths of this study is the diverse viewpoints obtained by enrolled participants, providing a "360 degree" view of the CAR T-cell therapy process in Aotearoa New Zealand – that is, not only patients, but support persons, clinicians and administrators. We intentionally enrolled patients and support persons with experience of both positive and negative outcomes after CAR T-cell therapy, as well as participants with a range of ages and ethnic backgrounds. This diversity of viewpoint, we feel, improved our ability to obtain a broad range of data on current experience, and helped us to identify new solutions.

Another strength of our study is the wide range of topics covered relating to CAR T-cell therapy. The flexibility of semi-structured interviews, as well as the wide variety of associations of participants to CAR T-cell therapy, means that this project considered many different facets of the treatment pathway, from the individual (e.g. patient and support person experience and communication between clinicians) to the system-level (identifying areas of inequity, and how a future service should be designed). This data-driven approach, as opposed to a theory-driven approach, meant that the insights from participants could extend beyond the areas foreseen by the prespecified interview template.

This study was designed in discussion with Māori, and specifically aimed to recruit Māori participants, to inform ways a CAR T-cell service should be designed to improve

Māori health outcomes. Our findings highlight the need for further research regarding tikanga, or Māori custom, relating to CAR T-cell therapies, including the role and form of karakia (blessings) at the time of cell collection, administration and disposal. Our findings regarding the needs of support persons touch on the importance of a holistic approach to health outcome evaluation, which for Māori includes mental, spiritual and family/community wellbeing, as well as physical health (the Te Whare Tapa Whā model [19]). Such work should be conducted using a kaupapa Māori (Māori-led) approach, to ensure the design of a new health pathway actively addresses inequities in Indigenous health outcomes.

A limitation of this study is that by interviewing a wide group of participants, and having a semi-structured interview with scope to follow the interviewee to cover topics in more depth, data saturation was not reached in all areas. Indeed, given the limited numbers of New Zealanders who have received CAR T-cell therapy, data saturation may not have been possible. A further restriction to research into patient experience of relapsed/refractory non-Hodgkin lymphoma is that the experience of patients not responding to treatment may be lost due to rapid progression of their disease. While we attempted to ameliorate this by interviewing support persons, clinicians and administrators, this may have served to dilute the patient voice. We attempted to mitigate this by testing the thematic analysis within a participant workshop, to gain reflections on themes raised by others. We also note that the themes we have identified, including barriers to accessing CAR T-cell therapy, align with those identified elsewhere [3,6,8,13,20].

For peer review only

The focus of the interviews in this study was on the treatment experience and on areas of focus for future service design. While specific suggestions arose that might be incorporated into a future pathway (see Table 1, Global Theme 3), these suggestions alone are not sufficient to address all of the challenges that were identified, particularly with regards to equity. It is hoped that this work will help to identify areas for future focus; such research could include a more comprehensive identification of strategies to address the challenges raised.

While other studies have assessed the patient experience of CAR T-cell therapy via qualitative interviews [13,21], or assessed the principles required for a CAR T-cell service [8], our study is the first to examine this in Aotearoa New Zealand. This study emphasises the importance of co-design to elevate the voice of the indigenous Māori population, and to address the current severely limited access to CAR T-cell therapy. The suggestions unearthed in this study should help development of an intentionally designed service.

In summary, the RE-TELL study has defined the current experience of New Zealanders receiving CAR T-cell therapy; identified some of the barriers to those seeking treatment; and identified improvements for a future service, as well as areas for attention for those designing this service. Although our research is specific to Aotearoa New Zealand, elements of our findings are likely to apply to other countries intending to develop CAR T-cell therapy services, particularly those countries in which indigenous people experience health inequities.

For peer review only

ACKNOWLEDGEMENTS

The authors would like to thank the participants for their time and insights, and for their willingness to share their experiences. We acknowledge those who have passed, and uphold this study as part of their legacy. We also acknowledge Myra Ruka for input into design of the study and review of the manuscript. This study was presented in a provisional form at the Blood Conference 2022.

FUNDING

This study was supported by funding from Janssen-Cilag Pty Ltd to Deloitte and the Malaghan Institute (grant: N/A). RW is supported by a Clinical Practitioner Fellowship from the Health Research Council (19/139). RF is supported by the LifeBlood Trust. The Malaghan Institute acknowledges philanthropic funding support from David Downs and The Thompson Family Foundation.

COMPETING INTERESTS

RF, RW and DS are employees of the Malaghan Institute of Medical Research, which received funding from Janssen-Cilag Pty Ltd towards the cost of this research. OA, KK and MJ are employees of Deloitte, which received funding from Janssen-Cilag Pty Ltd through a contract for services. Janssen-Cilag had no involvement in the conduct of the research, data collection, data analysis, or in preparation or approval of the manuscript.

CONTRIBUTORSHIP STATEMENT

RF, OA and RW designed the study and wrote the study protocol. RF, OA, KK and MJ performed study procedures. RF, OA, DS and RW analysed the data and drafted the manuscript. All authors reviewed and approved the final manuscript.

For peer review only

DATA SHARING STATEMENT

Given the commitments made to participants regarding deidentification of data shared from this research, the authors are not able to share participant transcripts from interviews.

ETHICS APPROVAL STATEMENT

Ethical approval for this study was provided by Victoria University of Wellington Human Ethics Committee (ref: 29982) before any study activities were commenced. All participants gave written informed consent.

For peer review only

REFERENCES

1. Locke F, Miklos D, Jacobson C et al. Axicabtagene Ciloleucel as Second-Line Therapy for Large B-Cell Lymphoma. *N Engl J Med* 2022; 386: 640-654
2. Schuster S, Bishop M, Tam C et al. Tisagenlecleucel in Adult Relapsed or Refractory Diffuse Large B-Cell Lymphoma. *N Engl J Med* 2019; 380:45-56.
3. Jommi C, Bramanti S, Pani M et al. CAR T-Cell Therapies in Italy: Patient Access Barriers and Recommendations for Health System Solutions. *Front. Pharmacol* 2022; 13:915342.
4. Lim F, Yunxin C, Huang FJ et al. A unique hub-and-spoke model to optimize patient management in lymphoma using novel CAR T-cell therapy in southeast and south Asia. *Haematol Oncol* 2022; doi: 10.1002/hon.3057. Online ahead of print.
5. Hudson M, Mead A, Chagné D et al. Indigenous Perspectives and Gene Editing in Aotearoa New Zealand. *Front Bioeng. Biotechnol* 2019; 7: 70.
6. Kamal-Bahl, Puckett J, Bagchi I et al. Barriers and solutions to improve access for chimeric antigen receptor therapies. *Immunotherapy* 2022; doi: 10.2217/imt-2022-0037. Online ahead of print.
7. Emole J, Lawal O, Lupak O et al. Demographic differences among patients treated with chimeric antigen receptor T-cell therapy in the United States. *Cancer Medicine* 2022; doi: 10.1002/cam4.4797. Online ahead of print.
8. Gagelmann N, Sureda A, Montonto S et al. Access to and affordability of CAR T-cell therapy in multiple myeloma: an EBMT position paper. *Lancet Haemtol* 2022; 9:e786-95.
9. George P, Dasyam N, Giunti G et al. Third-generation anti-CD19 chimeric antigen receptor T-cells incorporating a TLR2 domain for relapsed or refractory B-cell lymphoma: a phase I clinical trial protocol (ENABLE). *BMJ Open* 2020; 10(2):e034629.
10. Gurney J, Stanley J, McLeod M et al. Disparities in cancer-specific survival between Māori and non-Māori New Zealanders, 2007-2016. *JCO Glob Oncol* 2020; 6:766-774.
11. Ministry of Health. New Zealand Cancer Action Plan 2019-2029 – Te Mahere mō te mate Pukupuku o Aotearoa 2019-2029. *Ministry of Health* 2020.
12. Zozaya N, Villaseca J, Abdalla F et al. A strategic reflection for the management and implementation of CAR-T therapy in Spain: an expert consensus paper. *Clin Transl Oncol* 2022; 24(6):968-980.
13. Castillo G, Lalu M, Asad S et al. Navigating choice in the face of uncertainty: using a theory informed qualitative approach to identifying potential patient barriers and enablers to participating in an early phase chimeric antigen receptor T (CAR-T) cell therapy trial. *BMJ Open*. 2021; 11(3):e043929.
14. Chow R, Bradley E, Gross C. Comparison of Cancer-Related Spending and Mortality Rates in the US vs. 21 High-Income Countries. *JAMA Health Forum*. 2022; 3(5):e221229.
15. Weinkove R, George P, Ruka M et al. Chimeric antigen receptor T-cells in New Zealand: challenges and opportunities. *N Z Med J* 2021; 134(1542):96-108.
16. Boyd H, McKernon S, Mullin B et al. Improving healthcare through the use of co-design. *N Z Med J* 2012; 125(1357):76-87.
17. Braun V, Clarke V. Using thematic analysis in psychology. *Qualitative Research in Psychology* 2006; 3(2):77-101.
18. Fugard A, Potts H. Thematic analysis. *SAGE Research Methods Foundation* 2019; https://dx.doi.org/9781529748659.

For peer review only

19. Durie M. Whaiora: Maori Health Development. *Auckland: Oxford University Press* 1998; pp68-74.
20. Buradagunta C, Garacci Z, D'Souza A et al. Socioeconomic disadvantage contributes to ethnic disparities in multiple myeloma survival: a matched cohort study. *Blood Cancer Journal* 2022; 12:82.
21. Shah N, Delforge M, San-Miguel J et al. Patient experience before and after treatment with idecabtagene vicleucel (ide-cel, bb2121): qualitative analysis of patient interviews in the KarMMa trial. *Leuk Res* 2022; 120:106921.

For peer review only

Supplementary file 1: Semistructured interview guide

For patients and support persons

Introduction and explanation of session
Entry questions

- Age
- Ethnicity
- Education
- Whether received treatment or supported someone through treatment

Experiences through treatment

- Describe your CAR T-cell treatment
  - When did you first hear about CAR T-cells?
  - How did you travel to obtain CAR T-cells?
  - How did you find the treatment process?
    - How long did you spend in another city obtaining CAR T-cell treatment?
  - How did you find returning to your home city after treatment?
    - How well did your different health professionals co-ordinate together?
    - How did you find the follow-up period after having treatment with CAR T-cells?
- What was the emotional impact of the treatment?
  - Were your expectations met by the treatment?
  - Is there anything that could have made the treatment easier for you?
- What was the impact of your treatment on your support person and wider whānau/family?
- What was the financial impact of your treatment?
  - What support was provided during this time?
  - What other supports could have helped?

Treatment aspects that worked well

- What parts of your treatment journey do you think worked well?
- What parts of your treatment should be preserved, or kept, if more people were treated with CAR T-cells in the future?

Treatment aspects that could be improved upon

- What parts of your treatment do you think could be improved upon?

- How could the process of travelling to-and-from the CAR T-cell treating centre be improved?
- What parts of your medical treatment did you find most challenging?
- Were the explanations about CAR T-cells accurate for your experience?
  - If not, why not?

For peer review only

  - What else would you have wanted to know about before receiving CAR T-cell therapy?
- Were there any emotional supports that could have made this process easier for you?
- What challenges did you face in accessing CAR T-cells that you think could be avoided in future?
- Did you feel your culture was respected throughout your treatment?
  - If not, why not?
- Did you feel your religion was respected throughout your treatment?
  - If not, why not?
- What challenges did you face in accessing CAR T-cell treatment?
  - How could this be addressed in future?

For peer review only

For clinicians and administrators

Introduction and explanation of session
Entry questions

- Age
- Ethnicity
- Education
- Role in CAR T-cell treatment
- Experience with CAR T-cell treatment

System flow

- How well does the current healthcare system facilitate access to CAR T-cell therapy?
- How efficient do you think the current system is?
- What are the current barriers to patients accessing CAR T-cell treatment?
- What are the current barriers to patients moving through CAR T-cell treatment?
- What factors help patients access CAR T-cell treatment?
- Do you think current access to CAR T-cell therapy is equitable?
    - If not, what are the main current barriers?
        - How could these be addressed?

Staff experience of the CAR T-cell therapy pathway

- Did you have all the information you needed to help your patient access CAR T-cell treatment?
    - What else would you have wanted to know prior to the treatment?
- What resources could help improve the CAR T-cell treatment pathway from a practical perspective?
- What was your experience of the CAR T-cell treatment programme from an emotional perspective?
    - Are support resources needed for staff during this treatment?

For peer review only

Supplementary file 2: Visual representation of patient journey through CAR T-cell therapy

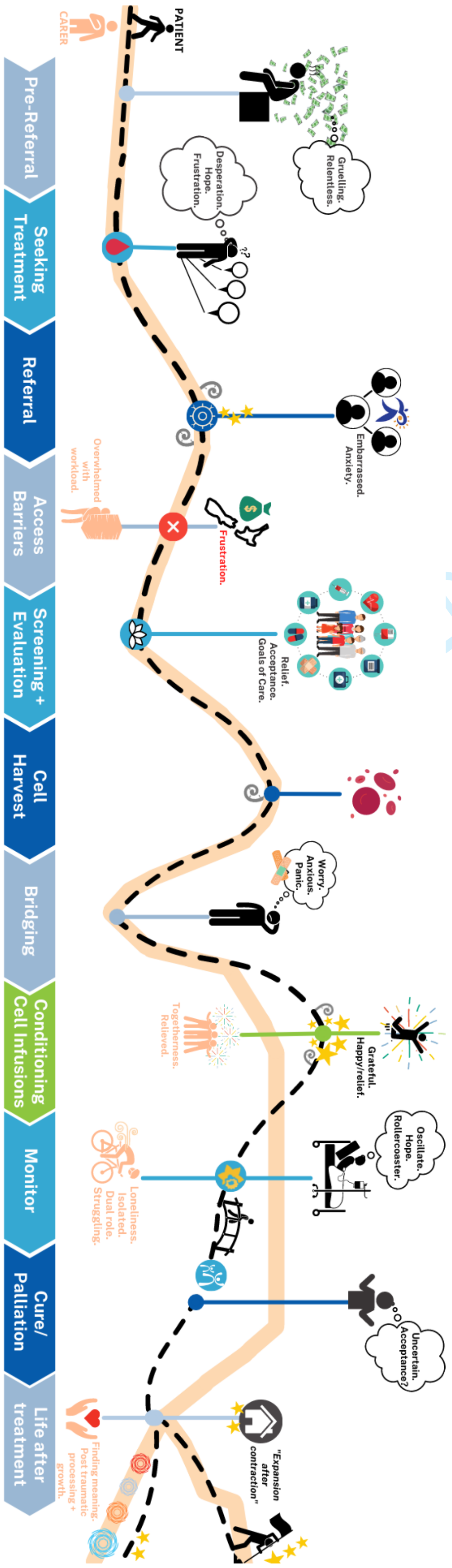

For peer review only

Supplemental file 3: Original RE-TELL study protocol

Full study title: REviewing patients and carers' experiences to inform the co-design of an optimal CAR T-cELL therapy service (RE-TELL study)

Protocol version: 1.0

Protocol date: 17 December 2021

Principal investigator: Robert Fyfe

Co-investigator:
Robert Weinkove
Olivia Anstis
Stephanie Easthope
Kushant Kapadia

For peer review only

1. Background and significance

Chimaeric antigen receptor (CAR) T-cell therapy is an emerging cellular therapy for the treatment of cancer. It involves using a patient's own T cells and modifying them with a CAR to both specifically target their cancer and then upregulate their immune system to eradicate the cancer. At this stage it is used primarily for blood cancers such as non-Hodgkin's lymphoma or B-acute lymphoblastic leukaemia, but the technique is being used to develop CAR T-cells to treat both other blood cancers such as myeloma as well as potentially solid cancers. This technique may have increasing use in the future to both use a new form of immunotherapy to treat cancers that do not respond to conventional chemotherapy, and also are in most cases more tolerable to patients than the untargeted approach of chemotherapy.

Current access to CAR T-cell therapy in New Zealand is limited to either: (a) government funding for standard-of-care CAR T-cells in exceptional circumstances, (b) private funding, or (c) through the ENABLE clinical trial, run by the Malaghan Institute of Medical Research. As access to this treatment is likely to improve in future, there is currently an opportunity to use the experience of New Zealanders who have received CAR T-cells to design how an optimal CAR T-cell care pathway rather than instituting a service in an ad-hoc fashion once funding is approved.

There are unique aspects to the clinical delivery of CAR T-cells, including the cell harvest, cell manufacture, and monitoring for toxicities and infection after cell delivery, which are different to other current treatments. There are also distinctive aspects to the New Zealand context, including the role of Māori engagement and kaitiakitanga (guardianship) in shaping new treatments , the equity focus, and a unique regulatory environment (Weinkove, 2021). Therefore, a treatment pathway tailor-made for New Zealand is needed, rather than translocating other system imprinting them on a New Zealand context.

Our aspiration is that the CAR T-cell treatment pathway designed through this work can then inform the design of CAR T-cell treatments delivered in New Zealand in the future.

2. Study hypothesis and aims

The hypothesis of this study is that the current treatment pathway for CAR T-cell therapy can be improved upon for future treatments by the involvement of both patients, support persons, clinicians and administrators in a co-design project.

For peer review only

The aim is to, by first interviewing stakeholders and then having focus groups for group discussion, produce an optimal treatment pathway for future CAR T-cell therapy to use.

3. Administrative organisation

Francis Health is an internationally networked health and disability consultancy firm, delivering strategic planning, service design, service improvement, research and evaluation across New Zealand, Australia and the United Kingdom.

Francis Health has operated for 19 years with a track record of successfully supporting healthcare organisations in New Zealand. The Francis Health NZ team has 15 staff with a range of expertise, both clinical and non-clinical. We have a large network of expert associates available to support programmes as needed.

4. Study design

Study population

At this point in time there are a very small number of adults in New Zealand who have undergone CAR T-cell therapy. The RE-TELL study team are aware of 11 adults, with there being potentially several others currently unbeknownst to the study team.

The inclusion criteria for patients and support persons are:
- Have undergone, or have supported someone with CAR-T therapy
- Aged 16-80 years
- Have access to a telephone or internet for the purposes of an interview
- Able to provide informed consent to participate in the study in accordance with ICH-GCP (International Conference on Harmonisation, Good Clinical Practice) principles

In addition, we will recruit five clinicians (including doctors, nurses and administrators) for interviews, and four administrators (including NGO providers, treatment co-ordinators, ward clerks and members of charitable trusts).

Inclusion criteria will be:
- Have been involved in the treatment of someone with CAR T-cell therapy in New Zealand
- Have access to a telephone or internet for the purposes of an interview
- Able to provide informed consent to participate in the study in accordance with ICH-GCP principles.

Sample size determination

For peer review only

The justification of sample size in the current qualitative study is based on the context of the research, and the availability of participants. It is understood that in qualitative research, narratives from as few as one participant can provide value (Boddy, 2016), and there is no set standard in terms of number of participants required to reach thematic saturation (Sandelowski, 1995). The context of this study is limited to the experience of patients who have undergone CAR-T therapy in New Zealand, and their carers. Currently only 11 patients have undergone CAR-T therapy in New Zealand. Therefore this study aims to recruit up to six patients and four support persons. In addition, we will aim to recruit five clinicians (Specialists, Registrars, Specialist Nurses (including Leukapheresis Nurse), Ward Nurse) and four healthcare administrators (NGO providers, treatment coordinators and ward clerks). This gives a total of 19 participants in the study.

5. Study procedures

Participant selection

We will use purposive sampling in order to enrol participants with a breadth of experience (treated in NZ vs. overseas; different cancers; different outcomes) and also breadth of backgrounds (age, ethnicity, gender, rural vs. urban). The intention will be to recruit in line with population parity with regards to age, ethnicity and gender; however given the small numbers of eligible participants and possible challenges with recruitment, it is not felt feasible to undertake representative sampling.

In addition, we will recruit five clinicians (including doctors, nurses and administrators) for interviews, and four administrators (including NGO providers, treatment co-ordinators, ward clerks and members of charitable trusts). Again, purposive sampling will be used to identify a breadth of experience and perspectives.

Recruitment

Patients and support persons will be recruited through several channels. Firstly, those who have undergone CAR T-cell therapy through the ENABLE clinical trial, who are to date the only patients to have received CAR T-cells in New Zealand, will be contacted and invited to participate. In addition, patient advocates and Leukaemia Blood and Cancer NZ will be contacted to identify patients and their

For peer review only

support persons who have received CAR T-cell therapy overseas, either via self-funding or government funding.

Clinicians and administrators will be approached by the researchers on the basis of the relevance of their role to the CAR-T care pathway. Each of these participants should play an active role in the prescription, administration and/or coordination of CAR-T therapy in New Zealand. This would include:

- Prescribing clinicians
- Referring clinicians
- Nursing staff (e.g. Nurse Specialist, Leukapheresis Nurse, Ward Nurse)
- Treatment/Study Coordinators
- NGO providers (e.g. Leukaemia and Blood Cancer Foundation)
- Administrative staff
- Ward clerk

Participant screening

Prospective participants will be initially contacted by the primary investigator to discuss possible interest in the study, and to provide general information including the participant information and consent form.

Given that the patient population may be vulnerable, having lived with a cancer diagnosis and undergone treatment as part of a clinical trial, follow-up contact following indication of interest to participate will be completed by a Health Psychologist registered with the New Zealand Psychologists' Board. Health Psychologists adhere to a strict code of ethics, which ensures that vulnerable persons are not coerced into participating in research. In addition, the psychologist will be able to assess whether the person is of sound mind to participate. Criteria would include any observable difficulties with memory, concentration, coherence, orientation, speech and attention. It is expected that there may be some limitations given the effects of treatment and the age of participants, and this will be taken into consideration during the screening process. Health psychologists are also attuned to changes in affect that may indicate

For peer review only

participant distress. If a participant is noted to be suffering undue distress as a result of participating, they will be asked whether or not they would like to continue.

Consent

Prospective participants will be contacted via phone or via e-mail. After a discussion of the project, a patient information and consent form will be either mailed or e-mailed. The participant will then either post back the consent form in an addressed envelope, or sign and scan the consent form back to the study team.

To ensure informed consent has been obtained, the clinician will screen for any patient vulnerabilities before the initial phone call. This might include known difficulties with language or cognition. The clinician will call patients and carers and ask if they consent to be contacted by a research coordinator from Francis Health. This coordinator will be responsible for sending out and receiving the PICF. By way of this being managed by an independent party, this reduces the risk of patients feeling obliged to participate, and more comfortable to decline.

The PICF will include an outline of the sorts of questions the participant will be asked during the course of their interview, and participation in any co-design workshops. The limits of protecting their identity from other study participants will be explained as follows:

- Their identity can be protected if they participate in the interview only, with all identifying data being removed from transcripts and resultant reports or publications;
- Their identity can only be partially protected if they choose to participate in the Co-Design workshop, as they will become known to the wider study group. Their identity will not however be shared beyond this group, and the same caveats as explained above will apply to their interview.

In addition, prior to each interview commencing, the interviewer (Health Psychologist) will review the PICF, ask if the participant has any questions, and confirm the participants consent. All participants

For peer review only

will be offered the opportunity for a support person(s)/whanau to be present during the interview and co-design workshop.

For any participants who experience undue distress as a result of participation in the study, they will be offered supportive counselling via the Cancer Society (patients and carers), or an EAP service (staff members). All participants will be given the contact details of the national helpline 1737.

Qualitative assessment

Interviews with patients and whanau will focus on their experiences with CAR-T therapy. Interview questions will follow the themes of:

- Experiences through treatment;
- Parts that worked well; and
- Parts that could be improved on

"Experiences" will include:

- Practical elements, such as access to treatment, healthcare system navigation, transport, accommodation and financial impact.
- Informational needs (e.g. was enough information given at the right time, in ways you could understand).
- Emotional experiences - what was felt emotionally throughout the journey. (e.g. What was the emotional impact of treatment? Were your expectations met? Is there anything that could help make this process easier for you?)
- Cultural elements: were cultural and/or religious protocols respected and enacted throughout the process. Were there barriers along the way? What could be improved?

Interviews with clinicians and administrators will focus on their experiences with CAR-T therapy, following the themes of:

- System flow:
  - How well the system they work within facilitates access to CAR-T therapy – subjective and objective appraisals;
  - Their perceptions of the efficiency of system flow, as patients move through the CAR-T treatment pathway;
  - Barriers and enablers

For peer review only

- Staff experience of the CAR-T pathway:
    - Practical needs – is there anything that staff need that could improve the treatment pathway;
    - Informational needs – do all staff get the information they need at the right points along the journey;
    - Emotional needs – what is their emotional experience of CAR-T therapy. What is needed in terms of support resources for staff.

The interviews will be audio-recorded and transcribed. Resultant transcripts will be themed following a framework provided by Braun and Clarke (2006). Results of the interviews will be used to design an optimal care pathway which will be produced visually.

All participants will then be invited to participate in an online workshop, to review the optimal pathway. They will be asked to give their feedback on the design, and iterate any solutions that have been offered. The workshop will be facilitated by a Health Psychologist, and include staff members from the Malaghan Institute as well as consenting patients and whānau. All participants will be offered the opportunity to have a support person with them. This workshop, or focus group, will have specific ground rules designed to ensure a safe environment for all participants.

This project will employ co-design methodology to develop a patient- and whanau-centred pathway of care. Co-Design is a quality improvement framework that prioritises partnerships with service users to receive feedback and redesign services to meet users' needs. Following the methodology of Boyd et al (2012), this project will follow the six main elements of:

1. Engage: Recruiting key stakeholders, including clinical staff from Malaghan, and patients and whānau experienced with CAR T-cell therapy
2. Plan: interviewing staff to map out the necessary steps in the treatment pathway ('standard care'), pain points, and areas for improvement
3. Develop: Identifying key elements to include on the pathway to optimise patient- and whānau-centred care.
4. Decide: Assembling staff, patients and whānau together (via an online platform), to decide on what improvements to implement from the key elements

For peer review only - http://bmjopen.bmj.com/site/about/guidelines.xhtml

5. Change: Map out the requirements for making these improvements.

6. Data Storage

Patient information and consent forms will be stored in a secure area of Francis Health e.g. locked filing cabinet. The study specific number for each participant will be written on these forms, providing a link between the study number and identifiers. Consent forms will be destroyed after five years.

All electronic material will be stored on password protected computers, either within the Francis Health team or in the custody of the primary investigatory. This will include recordings of interviews, transcripts of the interviews, and recordings/summaries of the focus group session. The interviews will be stored under a unique anonymised study identifier, to prevent the participant being identified in the event of a data breach.

7. Study termination

Timing of study termination

Recruitment of participants shall cease at the earliest of the following:

- Recruitment of six patients; four support persons; five clinicians; and four administrators or support staff;
- Or June 2022

Publication and presentation of results

We anticipate that the study results will be published in an international scientific and medical journal. No participant will be identifiable from any published or presented outcomes of this study.

In addition, there will be a final report produced to Janssen Pharmaceuticals, the study sponsor, regarding optimal pathway design, requirements to enable optimal pathway implementation and delivery, and recommended next steps.

For peer review only

Destruction of study data

Study data will be kept by either Francis Health or Robert Fyfe, and then safely destroyed in a confidential manner.

References

Weinkove R et al (2021). Chimeric antigen receptor T-cells in New Zealand: challenges and opportunities. NZMA. 134 (1542): 97-108.

Boddy, C. R. (2016). Sample size for qualitative research. *Qualitative Market Research: An International Journal*, *19*(4), 426–432. https://doi.org/10.1108/QMR-06-2016-0053

Boyd, H., Mckernon, S., Mullin, B., & Old, A. (2012). Improving healthcare through the use of co-design. *NZMJ*, *29*(125). http://journal.nzma.org.nz/journal/125-1357/xxxx/

Braun, V., & Clarke, V. (2006). Using thematic analysis in psychology. *Qualitative Research in Psychology*, *3*(2), 77–101. https://doi.org/10.1191/1478088706qp063oa

Sandelowski, M. (1995). Sample size in qualitative research. *Research in Nursing & Health*, *18*(2), 179–183. https://doi.org/10.1002/NUR.4770180211

For peer review only

SRQR Guidelines, assessed for RE-TELL study (Fyfe R, et al).

Table 1

Standards for Reporting Qualitative Research (SRQR)[a]

No.	Topic	Item
	**Title and abstract**	
S1	Title	Concise description of the nature and topic of the study Identifying the study as qualitative or indicating the approach (e.g., ethnography, grounded theory) or data collection methods (e.g., interview, focus group) is recommended
S2	Abstract	Summary of key elements of the study using the abstract format of the intended publication; typically includes background, purpose, methods, results, and conclusions
	**Introduction**	
S3	Problem formulation	Description and significance of the problem/phenomenon studied; review of relevant theory and empirical work; problem statement
S4	Purpose or research question	Purpose of the study and specific objectives or questions
	**Methods**	
S5	Qualitative approach and research paradigm	Qualitative approach (e.g., ethnography, grounded theory, case study, phenomenology, narrative research) and guiding theory if appropriate; identifying the research paradigm (e.g., postpositivist, constructivist/ interpretivist) is also recommended; rationale[b]
S6	Researcher characteristics and reflexivity	Researchers' characteristics that may influence the research, including personal attributes, qualifications/experience, relationship with participants, assumptions, and/or presuppositions; potential or actual interaction between researchers' characteristics and the research questions, approach, methods, results, and/or transferability
S7	Context	Setting/site and salient contextual factors; rationale[b]
S8	Sampling strategy	How and why research participants, documents, or events were selected; criteria for deciding when no further sampling was necessary (e.g., sampling saturation); rationale[b]
S9	Ethical issues pertaining to human subjects	Documentation of approval by an appropriate ethics review board and participant consent, or explanation for lack thereof; other confidentiality and data security issues
S10	Data collection methods	Types of data collected; details of data collection procedures including (as appropriate) start and stop dates of data collection and analysis, iterative process, triangulation of sources/methods, and modification of procedures in response to evolving study findings; rationale[b]
S11	Data collection instruments and technologies	Description of instruments (e.g., interview guides, questionnaires) and devices (e.g., audio recorders) used for data collection; if/how the instrument(s) changed over the course of the study
S12	Units of study	Number and relevant characteristics of participants, documents, or events included in the study; level of participation (could be reported in results)
S13	Data processing	Methods for processing data prior to and during analysis, including transcription, data entry, data management and security, verification of data integrity, data coding, and anonymization/deidentification of excerpts
S14	Data analysis	Process by which inferences, themes, etc., were identified and developed, including the researchers involved in data analysis; usually references a specific paradigm or approach; rationale[b]
S15	Techniques to enhance trustworthiness	Techniques to enhance trustworthiness and credibility of data analysis (e.g., member checking, audit trail, triangulation); rationale[b]
	**Results/findings**	
S16	Synthesis and interpretation	Main findings (e.g., interpretations, inferences, and themes); might include development of a theory or model, or integration with prior research or theory
S17	Links to empirical data	Evidence (e.g., quotes, field notes, text excerpts, photographs) to substantiate analytic findings
	**Discussion**	
S18	Integration with prior work, implications, transferability, and contribution(s) to the field	Short summary of main findings; explanation of how findings and conclusions connect to, support, elaborate on, or challenge conclusions of earlier scholarship; discussion of scope of application/ generalizability; identification of unique contribution(s) to scholarship in a discipline or field
S19	Limitations	Trustworthiness and limitations of findings

(*Table continues*)

(Continued)

No.	Topic	Item
	**Other**	
S20	Conflicts of interest	Potential sources of influence or perceived influence on study conduct and conclusions; how these were managed
S21	Funding	Sources of funding and other support; role of funders in data collection, interpretation, and reporting

[a]The authors created the SRQR by searching the literature to identify guidelines, reporting standards, and critical appraisal criteria for qualitative research; reviewing the reference lists of retrieved sources; and contacting experts to gain feedback. The SRQR aims to improve the transparency of all aspects of qualitative research by providing clear standards for reporting qualitative research.
[b]The rationale should briefly discuss the justification for choosing that theory, approach, method, or technique rather than other options available, the assumptions and limitations implicit in those choices, and how those choices influence study conclusions and transferability. As appropriate, the rationale for several items might be discussed together.

S1: Completed – page 1
S2: Completed – page 3
S3: Completed – page 6
S4: Completed – page 7
S5: Completed – page 8
S6: Completed – pages 9-10
S7: Completed – page 7
S8: Completed – page 7
S9: Completed – page 10
S10: Completed – pages 9-10
S11: Completed – page 10; supplemental file 1
S12: Completed – pages 11-12
S13: Completed – page 10; supplemental file 3
S14: Completed – page 10
S15: Completed – page 8
S16: Completed – pages 11-23
S17: Completed – pages 21-22
S18: Completed – pages 24-26
S19: Completed – page 28
S20: Completed – page 30
S21: Completed – page 30

For peer review only

Experiences and perspectives on chimeric antigen receptor (CAR) T-cell therapy among recipients, carers and referrers (RE-TELL): a qualitative study to inform CAR T-cell service design

Journal:	*BMJ Open*
Manuscript ID	bmjopen-2022-071112.R1
Article Type:	Original research
Date Submitted by the Author:	01-Nov-2023
Complete List of Authors:	Fyfe, Robert; Malaghan Institute of Medical Research; Victoria University of Wellington<br>Anstis, Olivia; Health Advisory, Deloitte Limited<br>Kapadia, Kushant; Health Advisory, Deloitte Limited<br>Jordan, Mallory; Health Advisory, Deloitte Limited<br>Sword, Danielle; Malaghan Institute of Medical Research<br>Weinkove, Robert; Malaghan Institute of Medical Research; Te Whatu Ora Health New Zealand Capital Coast and Hutt Valley, Te Rerenga Ora Blood & Cancer Centre
**Primary Subject Heading**:	Public health
Secondary Subject Heading:	Haematology (incl blood transfusion), Health services research
Keywords:	QUALITATIVE RESEARCH, HAEMATOLOGY, Organisation of health services < HEALTH SERVICES ADMINISTRATION & MANAGEMENT, Health Equity, Lymphoma < HAEMATOLOGY

SCHOLARONE™
Manuscripts

I, the Submitting Author has the right to grant and does grant on behalf of all authors of the Work (as defined in the below author licence), an exclusive licence and/or a non-exclusive licence for contributions from authors who are: i) UK Crown employees; ii) where BMJ has agreed a CC-BY licence shall apply, and/or iii) in accordance with the terms applicable for US Federal Government officers or employees acting as part of their official duties; on a worldwide, perpetual, irrevocable, royalty-free basis to BMJ Publishing Group Ltd ("BMJ") its licensees and where the relevant Journal is co-owned by BMJ to the co-owners of the Journal, to publish the Work in this journal and any other BMJ products and to exploit all rights, as set out in our licence.

The Submitting Author accepts and understands that any supply made under these terms is made by BMJ to the Submitting Author unless you are acting as an employee on behalf of your employer or a postgraduate student of an affiliated institution which is paying any applicable article publishing charge ("APC") for Open Access articles. Where the Submitting Author wishes to make the Work available on an Open Access basis (and intends to pay the relevant APC), the terms of reuse of such Open Access shall be governed by a Creative Commons licence – details of these licences and which Creative Commons licence will apply to this Work are set out in our licence referred to above.

Other than as permitted in any relevant BMJ Author's Self Archiving Policies, I confirm this Work has not been accepted for publication elsewhere, is not being considered for publication elsewhere and does not duplicate material already published. I confirm all authors consent to publication of this Work and authorise the granting of this licence.

For peer review only

TITLE

Experiences and perspectives on chimeric antigen receptor (CAR) T-cell therapy among recipients, carers and referrers (RE-TELL): a qualitative study to inform CAR T-cell service design

Authors

Fyfe R[1,2,3], Anstis O[4], Kapadia K[4], Jordan M[4], Sword D[1], Weinkove R[1,2,5].

Affiliations

1. Malaghan Institute of Medical Research, Wellington, New Zealand

2. Te Rerenga Ora Blood & Cancer Centre, Te Whatu Ora Health New Zealand – Capital, Coast and Hutt Valley, Wellington, New Zealand

3. Faculty of Science, Victoria University of Wellington, Wellington, New Zealand

4. Health Advisory, Deloitte Limited, Auckland, New Zealand.

5. Department of Pathology and Molecular Medicine, University of Otago Wellington, Wellington, New Zealand

Corresponding author

Dr Robert Fyfe

Email: Robert.Fyfe@ccdhb.org.nz

Telephone: +64 4 385 5999

Address: Te Rerenga Ora Blood & Cancer Centre, Te Whatu Ora Health New Zealand – Capital, Coast and Hutt Valley, 49 Riddiford Street, Wellington, New Zealand

Word, figure and reference counts

Abstract: 300 words

Article: 5062 words

Tables: 1

References: 21

For peer review only

Supplemental files: 3

For peer review only

ABSTRACT

Objectives:

RE-TELL is a qualitative study which aims to understand patient, support person, clinician and co-ordinator experiences and perspectives of chimeric antigen receptor (CAR) T-cell therapy, to inform design of a clinical CAR T-cell service in Aotearoa New Zealand.

Methods:

Qualitative interviews were undertaken with 19 New Zealand participants comprising: five CAR T-cell recipients, three support persons, six clinicians and five co-ordinators (including transplant co-ordinator, travel assistance, and charitable organisations). 4 participants identified as Māori. Semi-structured interviews focussed on domains of: experience through treatment; elements that work well, and those that could be improved upon. Interviews used thematic analysis to identify key themes. A workshop was held with participants to gain reflections on interim analysis and proposed improvements.

Results:

Thematic analysis identified three global themes. The first, “Sociocultural factors impact CAR T access”, identified potential sources of inequity including geographic, financial and informed consent barriers. The second, “Varying emotions, roles and enablers”, identified an easier treatment experience compared to alternatives; an underwhelming cell administration process; frustration with inpatient monitoring; burden on support persons; and importance of “bridge” organisations such as charities and patient support groups. Lastly, “Golden opportunities: re-imagining CAR T

service delivery", suggested: improved geographical access to CAR T-cell therapy, while retaining consolidated clinician experience; a 'dashboard' with information on CAR T-cell treatment, timeframes and manufacture; a health navigator to co-ordinate non-medical aspects of treatment and signpost care; embedding of indigenous data sovereignty and ownership of cells; a ceremony for cell infusion, incorporating family involvement and Māori cultural elements; and outpatient administration and monitoring where possible.

Conclusion:

This study has documented the current experience of New Zealanders receiving CAR T-cell therapy, and identified opportunities for future service development. These insights are relevant to service design both within Aotearoa New Zealand, and for other countries developing equitable CAR T-cell services.

STRENGTHS AND LIMITATIONS OF THIS STUDY

- Strengths
  - Diverse viewpoints pertaining to CAR T-cell therapy in Aotearoa New Zealand
  - Many facets of treatment experience explored
  - Specific focus on strategies to improve indigenous Māori health outcomes
- Limitations
  - Data saturation may not have been reached for all areas
  - Further research required to develop more solutions to existing challenges

For peer review only

INTRODUCTION

Chimeric antigen receptor (CAR) T-cell therapy has become a standard of care for some relapsed and refractory B-cell malignancies [1,2]. As a personalised cell and gene therapy involving gene modification of a patient's own T-cells, access to treatment is limited by logistical challenges and cost, while varied concerns regarding the technology need to be addressed, including equity of access, optimising the patient and support person experience, and cell and data sovereignty [3,4,5]. Internationally, CAR T-cell therapy provision has not consistently ensured equity of access [6,7], and equity has been highlighted as a key area of focus for ongoing development of this treatment modality [8].

Aotearoa New Zealand does not yet have a national service for standard of care CAR T-cell delivery, with access limited to clinical trial use [9] or overseas treatment. Within Aotearoa New Zealand, cancer-specific mortality is higher for indigenous Māori than for non-Māori, including for lymphoma and myeloma [10]. As a result, there is a strong focus on designing healthcare services to rectify this inequity [11].

While the challenges of delivering equitable access to CAR T-cell therapy have been considered worldwide [12,13], the Aotearoa New Zealand healthcare system has unique characteristics, such as: an imperative to promote indigenous health due to the government's obligations to Te Tiriti o Waitangi/the Treaty of Waitangi (the nation's founding document, which guarantees Māori the right to access to equitable healthcare and health outcomes); low public pharmaceutical funding compared to some high income countries [14]; and wide geographic dispersal [15].

For peer review only

We aimed to assess the current experiences of CAR T-cell therapy for patients, support persons, clinicians and administrators within Aotearoa New Zealand, to develop insights into treatment experience, needs, and barriers to treatment; and to identify potential improvements for future services. Our overall goal was to inform design of a future national CAR T-cell service. We conducted semi-structured qualitative interviews, assessed these interviews with thematic analysis, and further refined this analysis through a co-design workshop with participants, where the interim results of the thematic analysis were presented to participants for comment.

For peer review only

METHODS

Study design

In the RE-TELL study we recruited a total of 19 participants, including 5 patients, 3 support persons, 6 clinicians and 5 administrators, to provide a diversity of perspectives on CAR T-cell therapy. At the time of recruitment, there were an estimated 11 New Zealand patients who had received CAR T-cell therapy, given the limitations of research into a novel therapy for non-Hodgkin lymphoma. Of these, 7 were invited to participate (and accepted), wth the goal of recruiting 5 participants to provide a range of perspectives. 2 patients then became unable to participate due to health or logistical reasons. We also intended to recruit 5 support persons, clinicans and administrators, to provide additional perspectives while not prejudicing the patient voice. Participants were selected using purposive sampling to encompass a range of experiences (treated in Aotearoa New Zealand or overseas; different outcomes after treatment; different roles in a patient's care) and a diversity of backgrounds (age; ethnicity; gender; urban and rural). They were contacted by the primary investigator by e-mail with an invitation to participate and general information regarding the study.

A co-design methodology was chosen to take a partnership approach with patients and support persons, to understand patient experience, and to use this to develop potential improvements. Following the methodology of Boyd et al [16], the project followed the six main elements of: Engage, Plan, Explore, Develop, Decide, Change. Engage refers to establishing relationships with patients and staff to improve health services. Plan involves generating ideas about the goals of improvement work and strategies to achieve it. Explore refers to understanding patient and staff experiences, to identify

For peer review only

areas for improvement. Develop involves turning ideas into specific improvements. Decide involves a choice between improvements and how to implement them. Change means then turning ideas into action.

Data collection and analysis

Utilising semi-structured interviewing techniques, we guided participants along the treatment pathway, enquiring of:treatment experience; elements that worked well; elements that could be improved on; practical needs; informational needs; emotional experiences; cultural elements; and system flow. With a deductive approach in mind, this aimed to provide a logical framework with which to structure themes and experiences, and for participants to review themes and work towards service improvements. The full interview guide is provided as Supplemental Appendix 1. Interview data were analysed using thematic analysis, with transcripts themed using the above mentioned theoretical/deductive approach. Following the framework provided by Braun and Clarke [17], the following six steps were taken to analyse the data:

1. **Familiarisation with the data:** All interviews and the focus group were transcribed and independently reviewed by RF and OA. Participants were sent their own transcripts prior to analysis, so that they were able to read through and make corrections or add clarifications. The transcripts were referred to repeatedly throughout the analysis to understand and clarify meaning and resultant themes.
2. **Initial Coding.** Labels and tags were assigned within the transcripts, to identify information that related to phases of the treatment journey.
3. **Generating Initial Themes.** Coding was independently organised into practical, emotional, or cultural themes across the treatment pathway. New or novel information was highlighted to discuss between the researchers at the next phase.

For peer review only

4. **Reviewing and Defining themes.** RF and OA presented their coding and initial themes to each other, for agreement on global themes and subthemes.
5. **Data Charting:** Themes were mapped across the treatment pathway into multiple streams in a chart highlighting the experience of clinicians, patients and whānau.
6. **Reviewing and Refining themes.** Resultant themes were presented to participants to ratify and clarify in a focus group, and as a basis to form discussion on service improvements. These are the themes presented in this report.

All interviews and the workshop were facilitated by a Health Psychologist (OA) registered with the New Zealand Psychologist's Board. Interviews were performed between 13 January 2022 and 8 April 2022. Interviews were conducted via videoconference, as COVID-19 pandemic-related restrictions precluded in-person interviews. Interviews were audio-recorded, and lasted between 28 and 60 minutes. Interviewees were given the option to review manuscripts and provide comment.

A focus group was held to review and refine themes with participants (n = 7) who self-selected to attend a videoconference workshop, in order to gain reflections on the responses and potential service improvements. During this workshop, a graphical representation of the patient journey through CAR T-cell therapy was displayed, as described by RE-TELL participants (Supplementary appendix 2). This served to summarise information gathered during the interviews, to provide logical structure to the themes by mapping them across the treatment timeline, and to help overcome health literacy barriers. The responses from workshop participants assisted with the formulation of "Table 1, Global Theme 3: Future improvements to CAR T-cell pathway" and the improvements emphasised in the discussion; no new themes were

For peer review only

identified during the workshop. The primary themes from this workshop are presented in Results – Co-Design Workshop.

Interviews were transcribed verbatim and analysed using data analysis software NVivo V.11. Transcripts were analysed and coded independently into key themes by RF and OA according to existing guidelines [17, 18]. OA had no prior involvement with the participants or CAR T-cell therapy; RF was the clinical fellow for the ENABLE trial during the study, with clinical involvement with 2 patients and 1 participant. The researchers then met to seek alignment and generate global and sub-themes collaboratively. Subthemes were allocated to provide contextual explanation of key themes, or describe less common yet important topics or emotional descriptors. Themes were formulated into a table including sample belief statements and sample quotes.

Statistical and ethical considerations

Ethical approval for this study was provided by Victoria University of Wellington Human Ethics Committee (ref: 29982) before any study activities. All participants gave written informed consent. The original study protocol is available as supplemental file 3.

Patient and public involvement

Patients were not involved in setting the research question or formation of the study protocol, but their responses directed the interviews, and provided the themes. We

For peer review only

intend to disseminate the results to study participants in a newsletter suitable for a non-specialist audience.

RESULTS

Baseline characteristics

We enrolled 19 participants, including 5 patients, 3 support persons, 6 clinicians and 5 administrators. 4 participants were Māori, 11 New Zealand European, 2 Asian and 1 other; 12 of 19 participants (63%) were female, 7 male. The highest level of education was secondary education for 4 participants, and tertiary education for 15.

Among the 5 patients interviewed, all had B-cell non-Hodgkin lymphoma and had received CAR T-cell therapy. Their median age was 48 years (range 23-59). Three had received investigational third generation CAR T-cells within the phase 1 'ENABLE' trial in Aotearoa New Zealand [9]; one had received standard of care axicabtagene ciloleucel (axi-cel) in Australia via a government funding mechanism; and one had received lisocabtagene autoleucel in the U.S.A within a clinical trial. Four of the five patients had a complete response to CAR T-cell therapy at the time of interview.

Of the 3 support persons, one had supported a patient through treatment via the ENABLE trial; one through treatment in Australia; and one through treatment in the U.S.A.

The clinicians interviewed comprised four clinical haematologists who had referred patients for CAR T-cell therapy, one palliative care nurse practitioner and one research

For peer review only

nurse. The administrators comprised a charitable services co-ordinator, a bone marrow transplant co-ordinator, a transport co-ordinator and two social workers.

Data saturation

Data saturation occurs when no additional themes emerge from the data. This was not reached in this study for all areas of analysis, and reasons for this are explored in the Discussion. Specifically, many of the subthemes mentioned in Global Theme 3 did not reach saturation, as there were a multitude of potential areas improvements raised, which were not discussed with all participants.

Interviews: Key domains and themes:

Themes arising from the interviews were organized into three global themes, before subclassification into 8 domains and 23 subthemes (see Table 1). The first global theme, Sociocultural factors impact CAR T access, relates to both the challenges that patients and clinicians face in attempting to access CAR T-cell therapy, and the barriers and inequities that may prevent others from accessing the treatment. The second global theme, Varying emotions, roles and enablers, describes the experience of treatment within New Zealand or overseas, including the ancillary services in place to support patients. The final global theme, Golden opportunities: re-imagining CAR T service delivery, explores the ways that a future national CAR T-cell therapy service might improve treatment delivery.

For peer review only

1

Table 1: Global themes, key domains, subthemes and sample quotes		
Relevant domains	Subtheme	Sample quotes
**Global theme 1: Sociocultural factors impact CAR T access**		
Equity	Potential inequity stemming from lack of access	"To be equitable, you need to make sure it is accessible to everyone, and we can't just rely on clinical trials and compassionate access programmes" [C1].
	Geographic barriers	"The first barrier is distance" [C1]<br>"I understand you can't have CAR T in all centres. I just think one is probably not enough for many patients" [C1]<br>"You are asking someone who has a limited support network to fly… it's hard for the patient psychologically"[C1]
	Informed consent	"As soon as you start talking about how risky things are – a Māori worldview is often quality over quantity…. In a medicolegal worldview, we go into the nth degree about every small little potential risk, such that [Māori] will start to get scared and go well, actually, I don't want this." [C5]<br>"You should give me information that I can just hand to my mother and she understands it" [P1]
	Financial barriers	"People just don't have enough money… for financial support, transitioning from being a full time employee to having to stop." [A4]<br>"We say to patients we have this intensive treatment – it's fully funded, all public… but we have no mechanism to help you stay down here, and you've got to a charity [for accommodation]" [A2]
	Awareness of trial	"At least a couple of patients initiated the conversation about wanting to be part of the CAR T-cell trial" [C4]<br>"People don't know where to look sometimes" [C6]
	Requirement for advocacy	"Our health system requires you to navigate it yourself, it's so patient unfriendly" [P1]
**Global theme 2: Varying emotions, roles and enablers**		
Patient experience	Underwhelming administration of cells	"It's just a shot in your arm and then you're done… anti-climactic". [P5]
	Treatment experience easier compared to chemotherapy	"It was quite chill… chemo's a hell of a lot worse" [P5]<br>"A walk in the park" [P2]<br>"It was actually the first time through the whole cancer journey that I got to sleep a lot… I just rested" [P3]
	Frustration with inpatient monitoring	"I was so bored I went out to strip the bed, got some new sheets and made my bed up" [P2]
Support person experience	Emotional burden	"The relief to get him to the hospital and know that somebody else was looking after him, and it wasn't me that was having to keep things going every day" [S3]<br>"There's no care for the carers" [S1]

For peer review only

	Requirement to provide care for patients	"I felt like I'd gone from being a wife to a carer" [S3]
	Role in advocacy for patients	"Half the time I don't think [the patient] knew what day it was" [S3]
Bridges	Charities	"Sometimes patients and families do like to talk to an organization that understands, but is separate to the hospital… so they won't be judged" [A1]<br>"There is a period of time there where the hospital finishes their treating, and they may not be so unwell that a hospice is involved, but they're in that middle zone and it's really stressful." [A1]
	National Travel Assistance programme	"It's not meant to cover [all travel costs], but it's meant to … eliminate the barrier of those upfront costs" [A5]
	Patient support groups	"Show respect for what they've gone through and being able to articulate that experience to others" [A1]
Global theme 3: Golden Opportunities: re-imagining CAR T service delivery		
Centralised point of information	Accessible prepared resources	"It's good to have it delivered through a video. It's a bit easier to comprehend" [P1]<br>"Knowledge is power… if you've got the information and know what to expect, it takes a lot of the fear out of the process" [P3]
	Health navigator	"Someone who's thinking about the whole patient's life and experience going through the process, not just the medical side of it… a generalist, and then the specialists are doing their work". [P1]
Alterations to treatment delivery	Outpatient therapy	"I could have done a lot of that as an outpatient" [P2]
	Ceremony for cell infusion	Having family members, and potentially referring clinicians present for cell infusion: "It will show [patients] how important it is" [C5]
	Need for appropriate accommodation	"Really inappropriate [to require family members to sleep on the floor]" [P3]
Service design	Clinical staff experience	"You need to have [dedicated] staff because it's a unique therapy… [bone marrow transplant co-ordinators] couldn't just absorb [a CAR T-cell therapy role], they'd need extra resource". [A2]
Indigenous rights	Data sovereignty	"Is it information for information's sake, or is there a purpose at the end?" [C5]
	Cell sovereignty	"They told us what they were going to do with the stem cells if they didn't need them.. whereas in Australia? We never got those options". [S2]<br>"You almost want a courier tracking system [for cells]" [C5]

<u>Global theme 1: Accessing CAR T-cell therapy</u>

For peer review only - http://bmjopen.bmj.com/site/about/guidelines.xhtml

Potential inequity stemming from lack of access: Current mechanisms of access to CAR T-cell therapy in Aotearoa New Zealand are via clinical trial, self-funded treatment overseas, or government funded treatment overseas. There was broad agreement among those interviewed that current access to CAR T-cell therapy is inequitable, with the impression that those able to access treatment are likely to be more health literate, have more financial resources, are more likely to reside in an urban centre, and exist within societal structures that enable their health literacy and ability to navigate the health system.

Requirement for self-advocacy: Noting the barriers to accessing CAR T-cell therapy identified above, a frequently raised theme was that patients and support persons were required to advocate for themselves, either to access CAR T-cell therapy in the first instance, or to shape their clinical care to fit their needs. Although referrals for CAR T-cell therapy occurred

via a patient's haematologist/oncologist, several patients had to raise the option of CAR T-cell therapy with their treating clinician before this was considered. Many felt that the in the absence of standard-of-care CAR T-cell therapy in Aotearoa New Zealand, knowledge of referral pathways relies on 'word-of-mouth' communication.

Geographic barriers: Aotearoa New Zealand is a country with a low population density, long transport times between centres, and is distant from international centres that offer CAR T-cell therapies. Geographic inequity was identified as a factor impacting patients' ability to access therapy. Geographic barriers impacted people's finances and ability to care for their children, and resulted in time away from support networks and whānau (family), and concerns about housing and job security.

Informed consent: It was noted that many aspects of accessing treatments in Aotearoa New Zealand require an ability to navigate a complex medical system. In particular, the current process of consent for investigational treatments requires extensive cataloguing of possible risks of therapy. Participants commented that if this is provided without an equivalent explanation of potential benefits, patients may leave with the understanding that CAR T-cell therapies are riskier than they truly are (rather than accepting that the Western medicolegal paradigm requires robust documentation of each risk) [see: Table 1; global theme 2]. While this issue with informed consent was noted by both NZ European and Māori patients, it was thought that it may have greater impact on Māori patients. Interviewees noted that this could be further exacerbated by differences in health literacy and an inability for many clinicians to provide information within a Te Ao Māori (Māori world) framework, which could contribute to an erosion of trust in the health system.

Financial barriers: Although patients attempting to access CAR T via clinical trial in Aotearoa New Zealand experienced barriers, these were even more formidable for patients attempting to self-fund CAR T-cell therapy overseas. Among those accessing overseas treatment, patients and family were required to identify overseas hospitals with actively enrolling CAR T-cell therapy programmes; liaise with these hospitals and arrange transfer of medical information; fundraise high sums through combinations of personal funds, fundraising events or crowdfunding; and co-ordinate logistics to arrive at the treating hospital – all while unwell with their disease. Unsurprisingly, it was noted that many who considered exploring treatment via this route were eventually unable to access this.

For peer review only

Global theme 2: Varying emotions, roles and enablers

Treatment experience easier than chemotherapy: The predominant theme regarding the treatment experience was the relative tolerability of CAR T-cell therapy. New Zealanders who accessed CAR T-cells within the ENABLE trial did so as a last-line therapy, so had experience of numerous chemotherapy cycles, and often of stem cell transplantion. Participants reported that, although there were adverse effects, CAR T-cell therapy was much more comfortable and tolerable than their prior treatments. Indeed, the primary difficulty with CAR T-cell therapy was reported to be the prolonged inpatient monitoring (typically 14 days), or (for the recipient of axi-cel in Australia) monitoring in an Intensive Care Unit for cytokine release syndrome (CRS), while feeling well. Additionally, many of the treatment challenges highlighted were not primarily attributable to physical adverse events, but rather to socioeconomic factors. For those treated in Aotearoa New Zealand, these included: accessing suitable accommodation for patient and support person; affording groceries; and compensating for loss of income while a support person relocated to accompany the patient. For those treated outside Aotearoa New Zealand, these included: identifying overseas treating centres and securing a treatment place; complex and expensive logistical planning; and balancing having a support person at the treating centre to support the patient with maintaining family life at home. It was commented that conducting follow-up in an outpatient setting would have overcome many of the challenges of treatment (see Global Theme 3).

Underwhelming administration of cells: It was noted that the infusion of CAR T-cells could be anti-climactic, since it involved a very brief cell infusion. While some

For peer review only

patients found the moment of CAR T-cell administration momentous and significant, others felt it was underwhelming, and detracted from the treatment experience.

Support person experience: Our interviews identified the crucial role that support persons played in the care of patients receiving CAR T-cells. This included assistance with monitoring for CAR T-cell related adverse events, and co-ordinating the many non-medical challenges that arise as a patient goes through cancer treatment. In addition, support persons often played an active role by advocating for the patient. Reckoning with these new responsibilities, while managing the psychological stress of a close family member's life-threatening illness, presented a significant emotional burden for support persons.

Bridges: A characteristic of CAR T-cell therapy treatment for New Zealanders was the need for external organisations to serve as "bridges" to cover gaps in patient care that currently exist in the funded health system. These "bridges" include charitable organisations, such as Leukaemia and Blood Cancer NZ, who provide financial assistance for travel and accommodation costs during treatment, psychosocial support, and participation in support groups for patients to meet other patients receiving similar treatment. Such support groups also arise organically, such as via social media. Community hospices also provide a valuable service providing both end-of-life care and community outreach palliative care input.

The current healthcare system provides a National Travel Assistance programme, which covers a portion of the logistical and accommodation costs for patients and support persons travelling out-of-region for medical treatment. However, the gaps in

For peer review only

coverage of this programme result in costs falling to either patients or to charitable organisations.

Global theme 3: Golden opportunities: re-imagining CAR T service delivery

The final major theme related to improvement opportunities for a future national CAR T-cell therapy service.

Centralised point of information: It was recognized that the introduction of a CAR T-cell therapy should be accompanied by education and support structures to help clinicians and patients unfamiliar with the therapy to access it. For clinicians, this would require visible and accessible specialists to liaise with, and clear and accessible guidance of the referral process. For patients, this involves quality education materials to explain the treatment in understandable terms, preferably in video form in addition to text.

Health navigator: It was identified that a health navigator (sometimes referred to internationally as a "concierge") could serve a role as a patient liaison throughout treatment, coordinating both the medical and non-medical aspects of care (including logistic, financial and psychosocial considerations), providing patient advocacy, and education about aspects of treatment. An idiosyncratic element of CAR T-cell therapy is the cell manufacturing period, leading to a period of uncertainty for patients and referring clinicians as the outcome of CAR T-cell manufacture is awaited. Both clinicians and patients identified a potential role for a 'cell tracker', with which the progress of cell manufacture could be tracked over time, to manage expectations and provide reassurance.

For peer review only

Changes to treatment delivery: Several potential alterations to current CAR T-cell therapy clinical delivery were identified. First was the possibility of conducting a greater proportion (or even the entirety) of monitoring as an outpatient following cell infusion. This was felt to offer benefits for patient wellbeing as well as for health resourcing. Secondly, to address the underwhelming nature of cell administration, a ceremony for CAR T-cell administration was suggested, to imbue the procedure with a reverence befitting the occasion. This could involve family, friends or referring clinicians, either in person or via videoconference, as well as additional cultural or religious elements such as presence of kaumatua (Māori elders) and a karakia (blessing).

Dedicated clinical staff: It was identified that a CAR T-cell therapy service requires dedicated resourcing and training, rather than unfunded incorporation into overstretched bone marrow transplantation services. Treatment hubs should have dedicated CAR T-cell staff to allow a consolidation of skills and experience, and to ensure safe treatment delivery.

Indigenous sovereignty: Finally, it was identified that a future CAR T-cell service should include provisions to respect indigenous sovereignty over data and cells. For data, this should include anonymizing or de-identification of data shared beyond treating clinicians (unless explicit consent is given), as well as the understanding that data would lead to improvements for the patient group that the data is sourced from. For cells, this would involve explanation of where cells would be located, what processes would be undertaken with them, and what would happen to remaining cells

For peer review only

that are not required for their cellular therapy. As an example, one Māori participant underwent a cell collection procedure in Aotearoa New Zealand, before undergoing cell collection for a commercial CAR T-cell therapy overseas. They highlighted that in Aotearoa New Zealand they were informed about location of their cells during manufacture, and were given the option to either donate or dispose of cells that were not required for treatment. Conversely, during their treatment overseas they were not informed of the location of their cells or the manufacturing progress, nor given options about fate of their cells.

For peer review only

Co-design workshop

Participants noted that, for patients and support persons attempting to access CAR T-cell therapy, many of the challenges to be navigated are at or before the start of the pathway, and suggested refinements to the pre-referral process, and monitoring of referrals to ensure access is equitable.

Participants commented that, although many elements of the current pathway could be improved upon, including consent processes, support services and cultural elements, the primary focus of patients and support persons is to access CAR T-cell therapy, and that the need to improve these areas should not prevent initial adoption of the therapy.

Participants were asked to comment on which of the potential improvements (broadly identified in Global Theme 3) they would prioritise. This resulted in strong support for the health navigator role (to assist patients and families with non-medical and medical navigation of the health service and the effect that cancer treatment would have on their lives); the need for culturally appropriate care for all patients; and a centralised dashboard for information (to provide information about how to access CAR T-cell therapy and what treatment would entail). Elements such as a tracker for cells after extraction, and a ceremony around cell infusion, were considered improvements but less crucial to the success of a CAR T-cell therapy programme.

DISCUSSION

Aotearoa New Zealand has an unmet need for funded CAR T-cell therapies for B-cell non-Hodgkin lymphomas and other B-cell malignancies. As we expect CAR T-cell

For peer review only

therapy to become a standard of care in the future, it is important that consideration is given to the perspectives of patients, support persons, clinicians and administrators, in design of a national service. Therefore, we performed this qualitative study to generate insights into the current experiences of CAR T-cell therapy, and the areas which could be improved upon.

Elements of the current service were praised by participants. First, CAR T-cell therapy was considered more tolerable than other treatments such as chemotherapy and autologous stem cell transplantation. Second, the role of charities and other organisations in expanding on the support provided by healthcare providers, and mitigating the challenges facing patients and support persons through treatment was applauded. Third, the use of strategies to respect tissue sovereignty within the ENABLE trial was thought to lay important groundwork for further research in this area in future.

We identified several areas for potential improvement of CAR T-cell delivery. First were strategies to improve communication with patients and referring clinicians, and therefore minimise some of the barriers to access identified in Table 1. Some patients had to ask to be referred for the CAR T-cell clinical trial, suggesting that a more robust system of informing clinicians of open clinical trials is required. For a standard of care service, strategies could include a centralised 'dashboard' for new CAR T referrals (with clear guidance for clinicians on eligibility, process and timeframes), and high-quality educational materials for patients and family in understandable language. Communication could also be improved with a 'tracker' of cell collection and manufacture, which would provide patients and referring clinicians with an insight into timeframes.

For peer review only

Other improvements to improve the treatment experience included a transition to outpatient management, which would result in benefits for patients (improved psychosocial wellbeing and avoidance of infection while in hospital), alongside resourcing benefits (reduced inpatient burden, and less overall staff input). Such a transition would require more robust assistance from a National Travel Assistance programme, to ensure that treatment remains a realistic option for patients with fewer resources. A ceremony for CAR T-cell administration, including involvement of family and referring clinicians, and incorporating cultural or religious elements, may be appreciated by many patients.

Given the ethnic health disparities in Aotearoa New Zealand, including poorer cancer-specific survival for non-Hodgkin lymphoma and myeloma among Māori [10], a national CAR T-cell service will need to be designed with an explicit equity focus to ensure it improves Māori health outcomes and avoids replicating pre-existing issues with access to treatment and informed consent. Given the socioeconomic disparities since colonisation, this may require increased support to access treatment and to mitigate financial consequences for the patient and support persons relocating for treatment. It would also require culturally appropriate care within the CAR T-cell therapy team. Patient autonomy over data and cells was strongly valued, with the option to decide on how both are used.

Finally, we identified the need for treating hubs with consolidated experience and skills, to ensure that clinicians can deliver this new therapy safely. However, it was felt that keeping CAR T-cell therapy in a single treating centre, even in a country with a

For peer review only

relatively small population such as Aotearoa New Zealand, could perpetuate existing geographic inequities. Therefore, consideration will need to be given in the medium term to balancing the relative trade-offs between efficiencies gained from consolidated care and the benefits to patients of care closer to home.

One of the key strengths of this study is the diverse viewpoints obtained by enrolled participants, providing a “360 degree” view of the CAR T-cell therapy process in Aotearoa New Zealand – that is, not only patients, but support persons, clinicians and administrators. We intentionally enrolled patients and support persons with experience of both positive and negative outcomes after CAR T-cell therapy, as well as participants with a range of ages and ethnic backgrounds. This diversity of viewpoint, we feel, improved our ability to obtain a broad range of data on current experience, and helped us to identify new solutions.

Another strength of our study is the wide range of topics covered relating to CAR T-cell therapy. The flexibility of semi-structured interviews, as well as the wide variety of associations of participants to CAR T-cell therapy, means that this project considered many different facets of the treatment pathway, from the individual (e.g. patient and support person experience and communication between clinicians) to the system-level (identifying areas of inequity, and how a future service should be designed). This data-driven approach, as opposed to a theory-driven approach, meant that the insights from participants could extend beyond the areas foreseen by the prespecified interview template.

For peer review only

This study was designed in discussion with Māori, and specifically aimed to recruit Māori participants, to inform ways a CAR T-cell service should be designed to improve Māori health outcomes. Our findings highlight the need for further research regarding tikanga, or Māori custom, relating to CAR T-cell therapies, including the role and form of karakia (blessings) at the time of cell collection, administration and disposal. Our findings regarding the needs of support persons touch on the importance of a holistic approach to health outcome evaluation, which for Māori includes mental, spiritual and family/community wellbeing, as well as physical health (the Te Whare Tapa Whā model [19]). Such work should be conducted using a kaupapa Māori (Māori-led) approach, to ensure the design of a new health pathway actively addresses inequities in Indigenous health outcomes.

A limitation of this study is that by interviewing a wide group of participants, and having a semi-structured interview with scope to follow the interviewee to cover topics in more depth, data saturation was not reached in all areas. Indeed, given the limited numbers of New Zealanders who have received CAR T-cell therapy, data saturation may not have been possible. A further restriction to research into patient experience of relapsed/refractory non-Hodgkin lymphoma is that the experience of patients not responding to treatment may be lost due to rapid progression of their disease. While we attempted to ameliorate this by interviewing support persons, clinicians and administrators, this may have served to dilute the patient voice. We attempted to mitigate this by testing the thematic analysis within a participant workshop, to gain reflections on themes raised by others. We also note that the themes we have identified, including barriers to accessing CAR T-cell therapy, align with those identified elsewhere [3,6,8,13,20]. We also acknowledge that applying a deductive approach to

For peer review only

thematic analysis may have restricted the reporting of themes amongst participants. Guiding participants along the treatment pathway by way of discussion was a strategy to assist in reducing distress (patients and whanau could anticipate the next step in questioning), and also to assist in the building of a re-imagined pathway. An alternative approach could have been to use an inductive approach, with a broader approach to questioning. This may have resulted in themes that did not necessarily emerge in this work. In fact, during the feedback hui, participants commented that the journey before seeking treatment, and after finishing treatment, was of equal importance to them; however, this was beyond the scope of this project.

The focus of the interviews in this study was on the treatment experience and on areas of focus for future service design. While specific suggestions arose that might be incorporated into a future pathway (see Table 1, Global Theme 3), these suggestions alone are not sufficient to address all of the challenges that were identified, particularly with regards to equity. It is hoped that this work will help to identify areas for future focus; such research could include a more comprehensive identification of strategies to address the challenges raised.

While other studies have assessed the patient experience of CAR T-cell therapy via qualitative interviews [13,21], or assessed the principles required for a CAR T-cell service [8], our study is the first to examine this in Aotearoa New Zealand. This study emphasises the importance of co-design to elevate the voice of the indigenous Māori population, and to address the current severely limited access to CAR T-cell therapy. The suggestions unearthed in this study should help development of an intentionally

For peer review only

designed service that results in health gains for Māori, and does not exacerbate health inequities.

In summary, the RE-TELL study has defined the current experience of New Zealanders receiving CAR T-cell therapy; identified some of the barriers to those seeking treatment; and identified improvements for a future service, as well as areas for attention for those designing this service. Although our research is specific to Aotearoa New Zealand, elements of our findings are likely to apply to other countries intending to develop CAR T-cell therapy services, particularly those countries in which indigenous people experience health inequities.

ACKNOWLEDGEMENTS

The authors would like to thank the participants for their time and insights, and for their willingness to share their experiences. We acknowledge those who have passed, and uphold this study as part of their legacy. We also acknowledge Myra Ruka for input into design of the study and review of the manuscript. This study was presented in a provisional form at the Blood Conference 2022.

FUNDING

This study was supported by funding from Janssen-Cilag Pty Ltd to Deloitte and the Malaghan Institute (grant: N/A). RW is supported by a Clinical Practitioner Fellowship from the Health Research Council (19/139). RF is supported by the LifeBlood Trust. The Malaghan Institute acknowledges philanthropic funding support from David Downs and The Thompson Family Foundation.

For peer review only

COMPETING INTERESTS

RF, RW and DS are employees of the Malaghan Institute of Medical Research, which received funding from Janssen-Cilag Pty Ltd towards the cost of this research. OA, KK and MJ are employees of Deloitte, which received funding from Janssen-Cilag Pty Ltd through a contract for services. Janssen-Cilag had no involvement in the conduct of the research, data collection, data analysis, or in preparation or approval of the manuscript.

CONTRIBUTORSHIP STATEMENT

RF, OA and RW designed the study and wrote the study protocol. RF, OA, KK and MJ performed study procedures. RF, OA, DS and RW analysed the data and drafted the manuscript. All authors reviewed and approved the final manuscript.

DATA SHARING STATEMENT

Given the commitments made to participants regarding deidentification of data shared from this research, the authors are not able to share participant transcripts from interviews.

ETHICS APPROVAL STATEMENT

Ethical approval for this study was provided by Victoria University of Wellington Human Ethics Committee (ref: 29982) before any study activities were commenced. All participants gave written informed consent.

For peer review only

REFERENCES

1. Locke F, Miklos D, Jacobson C et al. Axicabtagene Ciloleucel as Second-Line Therapy for Large B-Cell Lymphoma. *N Engl J Med* 2022; 386: 640-654
2. Schuster S, Bishop M, Tam C et al. Tisagenlecleucel in Adult Relapsed or Refractory Diffuse Large B-Cell Lymphoma. *N Engl J Med* 2019; 380:45-56.
3. Jommi C, Bramanti S, Pani M et al. CAR T-Cell Therapies in Italy: Patient Access Barriers and Recommendations for Health System Solutions. *Front. Pharmacol* 2022; 13:915342.
4. Lim F, Yunxin C, Huang FJ et al. A unique hub-and-spoke model to optimize patient management in lymphoma using novel CAR T-cell therapy in southeast and south Asia. *Haematol Oncol* 2022; doi: 10.1002/hon.3057. Online ahead of print.
5. Hudson M, Mead A, Chagné D et al. Indigenous Perspectives and Gene Editing in Aotearoa New Zealand. *Front Bioeng. Biotechnol* 2019; 7: 70.
6. Kamal-Bahl, Puckett J, Bagchi I et al. Barriers and solutions to improve access for chimeric antigen receptor therapies. *Immunotherapy* 2022; doi: 10.2217/imt-2022-0037. Online ahead of print.
7. Emole J, Lawal O, Lupak O et al. Demographic differences among patients treated with chimeric antigen receptor T-cell therapy in the United States. *Cancer Medicine* 2022; doi: 10.1002/cam4.4797. Online ahead of print.
8. Gagelmann N, Sureda A, Montonto S et al. Access to and affordability of CAR T-cell therapy in multiple myeloma: an EBMT position paper. *Lancet Haemtol* 2022; 9:e786-95.
9. George P, Dasyam N, Giunti G et al. Third-generation anti-CD19 chimeric antigen receptor T-cells incorporating a TLR2 domain for relapsed or refractory B-cell lymphoma: a phase I clinical trial protocol (ENABLE). *BMJ Open* 2020; 10(2):e034629.
10. Gurney J, Stanley J, McLeod M et al. Disparities in cancer-specific survival between Māori and non-Māori New Zealanders, 2007-2016. *JCO Glob Oncol* 2020; 6:766-774.
11. Ministry of Health. New Zealand Cancer Action Plan 2019-2029 – Te Mahere mō te mate Pukupuku o Aotearoa 2019-2029. *Ministry of Health* 2020.
12. Zozaya N, Villaseca J, Abdalla F et al. A strategic reflection for the management and implementation of CAR-T therapy in Spain: an expert consensus paper. *Clin Transl Oncol* 2022; 24(6):968-980.
13. Castillo G, Lalu M, Asad S et al. Navigating choice in the face of uncertainty: using a theory informed qualitative approach to identifying potential patient barriers and enablers to participating in an early phase chimeric antigen receptor T (CAR-T) cell therapy trial. *BMJ Open*. 2021; 11(3):e043929.
14. Chow R, Bradley E, Gross C. Comparison of Cancer-Related Spending and Mortality Rates in the US vs. 21 High-Income Countries. *JAMA Health Forum*. 2022; 3(5):e221229.
15. Weinkove R, George P, Ruka M et al. Chimeric antigen receptor T-cells in New Zealand: challenges and opportunities. *N Z Med J* 2021; 134(1542):96-108.
16. Boyd H, McKernon S, Mullin B et al. Improving healthcare through the use of co-design. *N Z Med J* 2012; 125(1357):76-87.
17. Braun V, Clarke V. Using thematic analysis in psychology. *Qualitative Research in Psychology* 2006; 3(2):77-101.
18. Fugard A, Potts H. Thematic analysis. *SAGE Research Methods Foundation* 2019; https://dx.doi.org/9781529748659.

19. Durie M. Whaiora: Maori Health Development. *Auckland: Oxford University Press* 1998; pp68-74.
20. Buradagunta C, Garacci Z, D'Souza A et al. Socioeconomic disadvantage contributes to ethnic disparities in multiple myeloma survival: a matched cohort study. *Blood Cancer Journal* 2022; 12:82.
21. Shah N, Delforge M, San-Miguel J et al. Patient experience before and after treatment with idecabtagene vicleucel (ide-cel, bb2121): qualitative analysis of patient interviews in the KarMMa trial. *Leuk Res* 2022; 120:106921.

For peer review only

Supplementary file 1: Semistructured interview guide

For patients and support persons

Introduction and explanation of session
Entry questions

- Age
- Ethnicity
- Education
- Whether received treatment or supported someone through treatment

Experiences through treatment

- Describe your CAR T-cell treatment
    - When did you first hear about CAR T-cells?
    - How did you travel to obtain CAR T-cells?
    - How did you find the treatment process?
        - How long did you spend in another city obtaining CAR T-cell treatment?
    - How did you find returning to your home city after treatment?
        - How well did your different health professionals co-ordinate together?
        - How did you find the follow-up period after having treatment with CAR T-cells?
- What was the emotional impact of the treatment?
    - Were your expectations met by the treatment?
    - Is there anything that could have made the treatment easier for you?
- What was the impact of your treatment on your support person and wider whānau/family?
- What was the financial impact of your treatment?
    - What support was provided during this time?
    - What other supports could have helped?

Treatment aspects that worked well

- What parts of your treatment journey do you think worked well?
- What parts of your treatment should be preserved, or kept, if more people were treated with CAR T-cells in the future?

Treatment aspects that could be improved upon

- What parts of your treatment do you think could be improved upon?

- How could the process of travelling to-and-from the CAR T-cell treating centre be improved?
- What parts of your medical treatment did you find most challenging?
- Were the explanations about CAR T-cells accurate for your experience?
    - If not, why not?

For peer review only

  - What else would you have wanted to know about before receiving CAR T-cell therapy?
- Were there any emotional supports that could have made this process easier for you?
- What challenges did you face in accessing CAR T-cells that you think could be avoided in future?
- Did you feel your culture was respected throughout your treatment?
  - If not, why not?
- Did you feel your religion was respected throughout your treatment?
  - If not, why not?
- What challenges did you face in accessing CAR T-cell treatment?
  - How could this be addressed in future?

For peer review only

For clinicians and administrators

Introduction and explanation of session
Entry questions

- Age
- Ethnicity
- Education
- Role in CAR T-cell treatment
- Experience with CAR T-cell treatment

System flow

- How well does the current healthcare system facilitate access to CAR T-cell therapy?
- How efficient do you think the current system is?
- What are the current barriers to patients accessing CAR T-cell treatment?
- What are the current barriers to patients moving through CAR T-cell treatment?
- What factors help patients access CAR T-cell treatment?
- Do you think current access to CAR T-cell therapy is equitable?
    - If not, what are the main current barriers?
        - How could these be addressed?

Staff experience of the CAR T-cell therapy pathway

- Did you have all the information you needed to help your patient access CAR T-cell treatment?
    - What else would you have wanted to know prior to the treatment?
- What resources could help improve the CAR T-cell treatment pathway from a practical perspective?
- What was your experience of the CAR T-cell treatment programme from an emotional perspective?
    - Are support resources needed for staff during this treatment?

For peer review only

Supplementary file 2: Visual representation of patient journey through CAR T-cell therapy

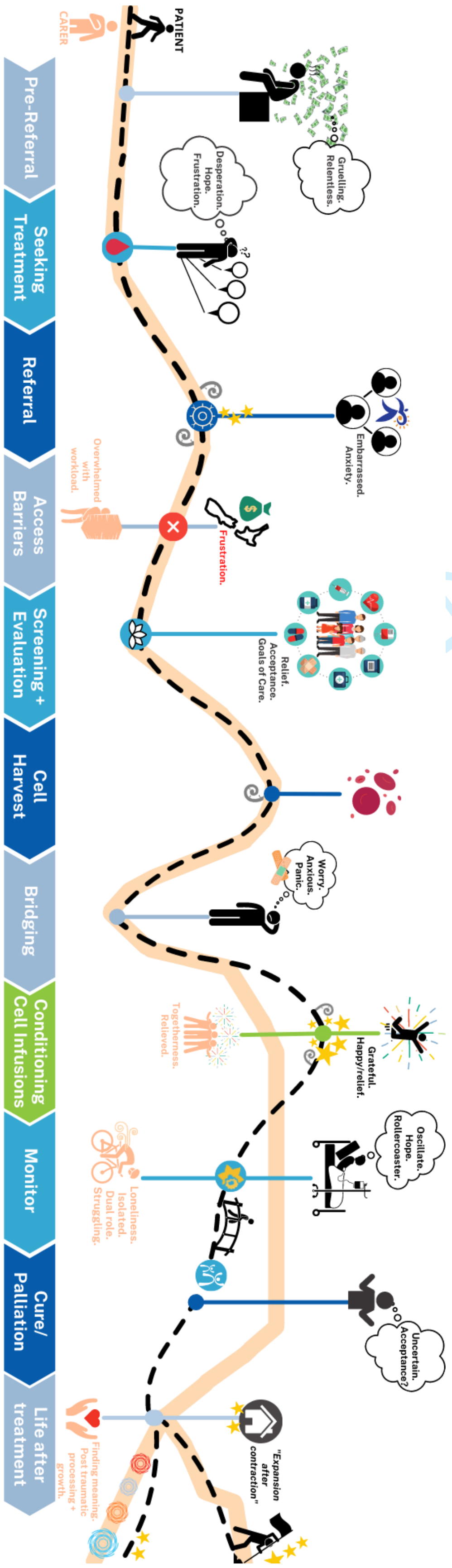

Supplemental file 3: Original RE-TELL study protocol

Full study title: REviewing patients and carers' experiences to inform the co-design of an optimal CAR T-cELL therapy service (RE-TELL study)

Protocol version: 1.0

Protocol date: 17 December 2021

Principal investigator: Robert Fyfe

Co-investigator:
Robert Weinkove
Olivia Anstis
Stephanie Easthope
Kushant Kapadia

For peer review only

1. Background and significance

Chimaeric antigen receptor (CAR) T-cell therapy is an emerging cellular therapy for the treatment of cancer. It involves using a patient's own T cells and modifying them with a CAR to both specifically target their cancer and then upregulate their immune system to eradicate the cancer. At this stage it is used primarily for blood cancers such as non-Hodgkin's lymphoma or B-acute lymphoblastic leukaemia, but the technique is being used to develop CAR T-cells to treat both other blood cancers such as myeloma as well as potentially solid cancers. This technique may have increasing use in the future to both use a new form of immunotherapy to treat cancers that do not respond to conventional chemotherapy, and also are in most cases more tolerable to patients than the untargeted approach of chemotherapy.

Current access to CAR T-cell therapy in New Zealand is limited to either: (a) government funding for standard-of-care CAR T-cells in exceptional circumstances, (b) private funding, or (c) through the ENABLE clinical trial, run by the Malaghan Institute of Medical Research. As access to this treatment is likely to improve in future, there is currently an opportunity to use the experience of New Zealanders who have received CAR T-cells to design how an optimal CAR T-cell care pathway rather than instituting a service in an ad-hoc fashion once funding is approved.

There are unique aspects to the clinical delivery of CAR T-cells, including the cell harvest, cell manufacture, and monitoring for toxicities and infection after cell delivery, which are different to other current treatments. There are also distinctive aspects to the New Zealand context, including the role of Māori engagement and kaitiakitanga (guardianship) in shaping new treatments , the equity focus, and a unique regulatory environment (Weinkove, 2021). Therefore, a treatment pathway tailor-made for New Zealand is needed, rather than translocating other system imprinting them on a New Zealand context.

Our aspiration is that the CAR T-cell treatment pathway designed through this work can then inform the design of CAR T-cell treatments delivered in New Zealand in the future.

2. Study hypothesis and aims

The hypothesis of this study is that the current treatment pathway for CAR T-cell therapy can be improved upon for future treatments by the involvement of both patients, support persons, clinicians and administrators in a co-design project.

For peer review only

The aim is to, by first interviewing stakeholders and then having focus groups for group discussion, produce an optimal treatment pathway for future CAR T-cell therapy to use.

3. Administrative organisation

Francis Health is an internationally networked health and disability consultancy firm, delivering strategic planning, service design, service improvement, research and evaluation across New Zealand, Australia and the United Kingdom.

Francis Health has operated for 19 years with a track record of successfully supporting healthcare organisations in New Zealand. The Francis Health NZ team has 15 staff with a range of expertise, both clinical and non-clinical. We have a large network of expert associates available to support programmes as needed.

4. Study design

Study population

At this point in time there are a very small number of adults in New Zealand who have undergone CAR T-cell therapy. The RE-TELL study team are aware of 11 adults, with there being potentially several others currently unbeknownst to the study team.

The inclusion criteria for patients and support persons are:
- Have undergone, or have supported someone with CAR-T therapy
- Aged 16-80 years
- Have access to a telephone or internet for the purposes of an interview
- Able to provide informed consent to participate in the study in accordance with ICH-GCP (International Conference on Harmonisation, Good Clinical Practice) principles

In addition, we will recruit five clinicians (including doctors, nurses and administrators) for interviews, and four administrators (including NGO providers, treatment co-ordinators, ward clerks and members of charitable trusts).

Inclusion criteria will be:
- Have been involved in the treatment of someone with CAR T-cell therapy in New Zealand
- Have access to a telephone or internet for the purposes of an interview
- Able to provide informed consent to participate in the study in accordance with ICH-GCP principles.

Sample size determination

The justification of sample size in the current qualitative study is based on the context of the research, and the availability of participants. It is understood that in qualitative research, narratives from as few as one participant can provide value (Boddy, 2016), and there is no set standard in terms of number of participants required to reach thematic saturation (Sandelowski, 1995). The context of this study is limited to the experience of patients who have undergone CAR-T therapy in New Zealand, and their carers. Currently only 11 patients have undergone CAR-T therapy in New Zealand. Therefore this study aims to recruit up to six patients and four support persons. In addition, we will aim to recruit five clinicians (Specialists, Registrars, Specialist Nurses (including Leukapheresis Nurse), Ward Nurse) and four healthcare administrators (NGO providers, treatment coordinators and ward clerks). This gives a total of 19 participants in the study.

5. Study procedures

Participant selection

We will use purposive sampling in order to enrol participants with a breadth of experience (treated in NZ vs. overseas; different cancers; different outcomes) and also breadth of backgrounds (age, ethnicity, gender, rural vs. urban). The intention will be to recruit in line with population parity with regards to age, ethnicity and gender; however given the small numbers of eligible participants and possible challenges with recruitment, it is not felt feasible to undertake representative sampling.

In addition, we will recruit five clinicians (including doctors, nurses and administrators) for interviews, and four administrators (including NGO providers, treatment co-ordinators, ward clerks and members of charitable trusts). Again, purposive sampling will be used to identify a breadth of experience and perspectives.

Recruitment

Patients and support persons will be recruited through several channels. Firstly, those who have undergone CAR T-cell therapy through the ENABLE clinical trial, who are to date the only patients to have received CAR T-cells in New Zealand, will be contacted and invited to participate. In addition, patient advocates and Leukaemia Blood and Cancer NZ will be contacted to identify patients and their

For peer review only

support persons who have received CAR T-cell therapy overseas, either via self-funding or government funding.

Clinicians and administrators will be approached by the researchers on the basis of the relevance of their role to the CAR-T care pathway. Each of these participants should play an active role in the prescription, administration and/or coordination of CAR-T therapy in New Zealand. This would include:

- Prescribing clinicians
- Referring clinicians
- Nursing staff (e.g. Nurse Specialist, Leukapheresis Nurse, Ward Nurse)
- Treatment/Study Coordinators
- NGO providers (e.g. Leukaemia and Blood Cancer Foundation)
- Administrative staff
- Ward clerk

Participant screening

Prospective participants will be initially contacted by the primary investigator to discuss possible interest in the study, and to provide general information including the participant information and consent form.

Given that the patient population may be vulnerable, having lived with a cancer diagnosis and undergone treatment as part of a clinical trial, follow-up contact following indication of interest to participate will be completed by a Health Psychologist registered with the New Zealand Psychologists' Board. Health Psychologists adhere to a strict code of ethics, which ensures that vulnerable persons are not coerced into participating in research. In addition, the psychologist will be able to assess whether the person is of sound mind to participate. Criteria would include any observable difficulties with memory, concentration, coherence, orientation, speech and attention. It is expected that there may be some limitations given the effects of treatment and the age of participants, and this will be taken into consideration during the screening process. Health psychologists are also attuned to changes in affect that may indicate

For peer review only

participant distress. If a participant is noted to be suffering undue distress as a result of participating, they will be asked whether or not they would like to continue.

Consent

Prospective participants will be contacted via phone or via e-mail. After a discussion of the project, a patient information and consent form will be either mailed or e-mailed. The participant will then either post back the consent form in an addressed envelope, or sign and scan the consent form back to the study team.

To ensure informed consent has been obtained, the clinician will screen for any patient vulnerabilities before the initial phone call. This might include known difficulties with language or cognition. The clinician will call patients and carers and ask if they consent to be contacted by a research coordinator from Francis Health. This coordinator will be responsible for sending out and receiving the PICF. By way of this being managed by an independent party, this reduces the risk of patients feeling obliged to participate, and more comfortable to decline.

The PICF will include an outline of the sorts of questions the participant will be asked during the course of their interview, and participation in any co-design workshops. The limits of protecting their identity from other study participants will be explained as follows:

- Their identity can be protected if they participate in the interview only, with all identifying data being removed from transcripts and resultant reports or publications;
- Their identity can only be partially protected if they choose to participate in the Co-Design workshop, as they will become known to the wider study group. Their identity will not however be shared beyond this group, and the same caveats as explained above will apply to their interview.

In addition, prior to each interview commencing, the interviewer (Health Psychologist) will review the PICF, ask if the participant has any questions, and confirm the participants consent. All participants

For peer review only

will be offered the opportunity for a support person(s)/whanau to be present during the interview and co-design workshop.

For any participants who experience undue distress as a result of participation in the study, they will be offered supportive counselling via the Cancer Society (patients and carers), or an EAP service (staff members). All participants will be given the contact details of the national helpline 1737.

Qualitative assessment

Interviews with patients and whanau will focus on their experiences with CAR-T therapy. Interview questions will follow the themes of:

- Experiences through treatment;
- Parts that worked well; and
- Parts that could be improved on

"Experiences" will include:

- Practical elements, such as access to treatment, healthcare system navigation, transport, accommodation and financial impact.
- Informational needs (e.g. was enough information given at the right time, in ways you could understand).
- Emotional experiences - what was felt emotionally throughout the journey. (e.g. What was the emotional impact of treatment? Were your expectations met? Is there anything that could help make this process easier for you?)
- Cultural elements: were cultural and/or religious protocols respected and enacted throughout the process. Were there barriers along the way? What could be improved?

Interviews with clinicians and administrators will focus on their experiences with CAR-T therapy, following the themes of:

- System flow:
  - How well the system they work within facilitates access to CAR-T therapy – subjective and objective appraisals;
  - Their perceptions of the efficiency of system flow, as patients move through the CAR-T treatment pathway;
  - Barriers and enablers

For peer review only

- Staff experience of the CAR-T pathway:
    - Practical needs – is there anything that staff need that could improve the treatment pathway;
    - Informational needs – do all staff get the information they need at the right points along the journey;
    - Emotional needs – what is their emotional experience of CAR-T therapy. What is needed in terms of support resources for staff.

The interviews will be audio-recorded and transcribed. Resultant transcripts will be themed following a framework provided by Braun and Clarke (2006). Results of the interviews will be used to design an optimal care pathway which will be produced visually.

All participants will then be invited to participate in an online workshop, to review the optimal pathway. They will be asked to give their feedback on the design, and iterate any solutions that have been offered. The workshop will be facilitated by a Health Psychologist, and include staff members from the Malaghan Institute as well as consenting patients and whānau. All participants will be offered the opportunity to have a support person with them. This workshop, or focus group, will have specific ground rules designed to ensure a safe environment for all participants.

This project will employ co-design methodology to develop a patient- and whanau-centred pathway of care. Co-Design is a quality improvement framework that prioritises partnerships with service users to receive feedback and redesign services to meet users' needs. Following the methodology of Boyd et al (2012), this project will follow the six main elements of:

1. Engage: Recruiting key stakeholders, including clinical staff from Malaghan, and patients and whānau experienced with CAR T-cell therapy
2. Plan: interviewing staff to map out the necessary steps in the treatment pathway ('standard care'), pain points, and areas for improvement
3. Develop: Identifying key elements to include on the pathway to optimise patient- and whānau-centred care.
4. Decide: Assembling staff, patients and whānau together (via an online platform), to decide on what improvements to implement from the key elements

For peer review only

5. Change: Map out the requirements for making these improvements.

6. Data Storage

Patient information and consent forms will be stored in a secure area of Francis Health e.g. locked filing cabinet. The study specific number for each participant will be written on these forms, providing a link between the study number and identifiers. Consent forms will be destroyed after five years.

All electronic material will be stored on password protected computers, either within the Francis Health team or in the custody of the primary investigatory. This will include recordings of interviews, transcripts of the interviews, and recordings/summaries of the focus group session. The interviews will be stored under a unique anonymised study identifier, to prevent the participant being identified in the event of a data breach.

7. Study termination

Timing of study termination

Recruitment of participants shall cease at the earliest of the following:

- Recruitment of six patients; four support persons; five clinicians; and four administrators or support staff;
- Or June 2022

Publication and presentation of results

We anticipate that the study results will be published in an international scientific and medical journal. No participant will be identifiable from any published or presented outcomes of this study.

In addition, there will be a final report produced to Janssen Pharmaceuticals, the study sponsor, regarding optimal pathway design, requirements to enable optimal pathway implementation and delivery, and recommended next steps.

For peer review only

Destruction of study data

Study data will be kept by either Francis Health or Robert Fyfe, and then safely destroyed in a confidential manner.

References

Weinkove R et al (2021). Chimeric antigen receptor T-cells in New Zealand: challenges and opportunities. NZMA. 134 (1542): 97-108.

Boddy, C. R. (2016). Sample size for qualitative research. *Qualitative Market Research: An International Journal*, *19*(4), 426–432. https://doi.org/10.1108/QMR-06-2016-0053

Boyd, H., Mckernon, S., Mullin, B., & Old, A. (2012). Improving healthcare through the use of co-design. *NZMJ*, *29*(125). http://journal.nzma.org.nz/journal/125-1357/xxxx/

Braun, V., & Clarke, V. (2006). Using thematic analysis in psychology. *Qualitative Research in Psychology*, *3*(2), 77–101. https://doi.org/10.1191/1478088706qp063oa

Sandelowski, M. (1995). Sample size in qualitative research. *Research in Nursing & Health*, *18*(2), 179–183. https://doi.org/10.1002/NUR.4770180211

For peer review only

SRQR Guidelines, assessed for RE-TELL study (Fyfe R, et al).

Table 1
Standards for Reporting Qualitative Research (SRQR)[a]

No.	Topic	Item
	**Title and abstract**	
S1	Title	Concise description of the nature and topic of the study Identifying the study as qualitative or indicating the approach (e.g., ethnography, grounded theory) or data collection methods (e.g., interview, focus group) is recommended
S2	Abstract	Summary of key elements of the study using the abstract format of the intended publication; typically includes background, purpose, methods, results, and conclusions
	**Introduction**	
S3	Problem formulation	Description and significance of the problem/phenomenon studied; review of relevant theory and empirical work; problem statement
S4	Purpose or research question	Purpose of the study and specific objectives or questions
	**Methods**	
S5	Qualitative approach and research paradigm	Qualitative approach (e.g., ethnography, grounded theory, case study, phenomenology, narrative research) and guiding theory if appropriate; identifying the research paradigm (e.g., postpositivist, constructivist/interpretivist) is also recommended; rationale[b]
S6	Researcher characteristics and reflexivity	Researchers' characteristics that may influence the research, including personal attributes, qualifications/experience, relationship with participants, assumptions, and/or presuppositions; potential or actual interaction between researchers' characteristics and the research questions, approach, methods, results, and/or transferability
S7	Context	Setting/site and salient contextual factors; rationale[b]
S8	Sampling strategy	How and why research participants, documents, or events were selected; criteria for deciding when no further sampling was necessary (e.g., sampling saturation); rationale[b]
S9	Ethical issues pertaining to human subjects	Documentation of approval by an appropriate ethics review board and participant consent, or explanation for lack thereof; other confidentiality and data security issues
S10	Data collection methods	Types of data collected; details of data collection procedures including (as appropriate) start and stop dates of data collection and analysis, iterative process, triangulation of sources/methods, and modification of procedures in response to evolving study findings; rationale[b]
S11	Data collection instruments and technologies	Description of instruments (e.g., interview guides, questionnaires) and devices (e.g., audio recorders) used for data collection; if/how the instrument(s) changed over the course of the study
S12	Units of study	Number and relevant characteristics of participants, documents, or events included in the study; level of participation (could be reported in results)
S13	Data processing	Methods for processing data prior to and during analysis, including transcription, data entry, data management and security, verification of data integrity, data coding, and anonymization/deidentification of excerpts
S14	Data analysis	Process by which inferences, themes, etc., were identified and developed, including the researchers involved in data analysis; usually references a specific paradigm or approach; rationale [b]
S15	Techniques to enhance trustworthiness	Techniques to enhance trustworthiness and credibility of data analysis (e.g., member checking, audit trail, triangulation); rationale[b]
	**Results/findings**	
S16	Synthesis and interpretation	Main findings (e.g., interpretations, inferences, and themes); might include development of a theory or model, or integration with prior research or theory
S17	Links to empirical data	Evidence (e.g., quotes, field notes, text excerpts, photographs) to substantiate analytic findings
	**Discussion**	
S18	Integration with prior work, implications, transferability, and contribution(s) to the field	Short summary of main findings; explanation of how findings and conclusions connect to, support, elaborate on, or challenge conclusions of earlier scholarship; discussion of scope of application/generalizability; identification of unique contribution(s) to scholarship in a discipline or field
S19	Limitations	Trustworthiness and limitations of findings

(*Table continues*)

(Continued)

No.	Topic	Item
	**Other**	
S20	Conflicts of interest	Potential sources of influence or perceived influence on study conduct and conclusions; how these were managed
S21	Funding	Sources of funding and other support; role of funders in data collection, interpretation, and reporting

[a]The authors created the SRQR by searching the literature to identify guidelines, reporting standards, and critical appraisal criteria for qualitative research; reviewing the reference lists of retrieved sources; and contacting experts to gain feedback. The SRQR aims to improve the transparency of all aspects of qualitative research by providing clear standards for reporting qualitative research.

[b]The rationale should briefly discuss the justification for choosing that theory, approach, method, or technique rather than other options available, the assumptions and limitations implicit in those choices, and how those choices influence study conclusions and transferability. As appropriate, the rationale for several items might be discussed together.

S1: Completed – page 1
S2: Completed – page 3
S3: Completed – page 6
S4: Completed – page 7
S5: Completed – page 8
S6: Completed – pages 9-10
S7: Completed – page 7
S8: Completed – page 7
S9: Completed – page 10
S10: Completed – pages 9-10
S11: Completed – page 10; supplemental file 1
S12: Completed – pages 11-12
S13: Completed – page 10; supplemental file 3
S14: Completed – page 10
S15: Completed – page 8
S16: Completed – pages 11-23
S17: Completed – pages 21-22
S18: Completed – pages 24-26
S19: Completed – page 28
S20: Completed – page 30
S21: Completed – page 30

For peer review only

BMJ Open

Experiences and perspectives on chimeric antigen receptor (CAR) T-cell therapy among recipients, carers and referrers (RE-TELL): a qualitative study to inform CAR T-cell service design

Journal:	*BMJ Open*
Manuscript ID	bmjopen-2022-071112.R2
Article Type:	Original research
Date Submitted by the Author:	17-Dec-2023
Complete List of Authors:	Fyfe, Robert; Malaghan Institute of Medical Research; Victoria University of Wellington<br>Anstis, Olivia; Health Advisory, Deloitte Limited<br>Kapadia, Kushant; Health Advisory, Deloitte Limited<br>Jordan, Mallory; Health Advisory, Deloitte Limited<br>Sword, Danielle; Malaghan Institute of Medical Research<br>Weinkove, Robert; Malaghan Institute of Medical Research; Te Whatu Ora Health New Zealand Capital Coast and Hutt Valley, Te Rerenga Ora Blood & Cancer Centre
**Primary Subject Heading**:	Public health
Secondary Subject Heading:	Haematology (incl blood transfusion), Health services research
Keywords:	QUALITATIVE RESEARCH, HAEMATOLOGY, Organisation of health services < HEALTH SERVICES ADMINISTRATION & MANAGEMENT, Health Equity, Lymphoma < HAEMATOLOGY

SCHOLARONE™
Manuscripts

I, the Submitting Author has the right to grant and does grant on behalf of all authors of the Work (as defined in the below author licence), an exclusive licence and/or a non-exclusive licence for contributions from authors who are: i) UK Crown employees; ii) where BMJ has agreed a CC-BY licence shall apply, and/or iii) in accordance with the terms applicable for US Federal Government officers or employees acting as part of their official duties; on a worldwide, perpetual, irrevocable, royalty-free basis to BMJ Publishing Group Ltd ("BMJ") its licensees and where the relevant Journal is co-owned by BMJ to the co-owners of the Journal, to publish the Work in this journal and any other BMJ products and to exploit all rights, as set out in our licence.

The Submitting Author accepts and understands that any supply made under these terms is made by BMJ to the Submitting Author unless you are acting as an employee on behalf of your employer or a postgraduate student of an affiliated institution which is paying any applicable article publishing charge ("APC") for Open Access articles. Where the Submitting Author wishes to make the Work available on an Open Access basis (and intends to pay the relevant APC), the terms of reuse of such Open Access shall be governed by a Creative Commons licence – details of these licences and which Creative Commons licence will apply to this Work are set out in our licence referred to above.

Other than as permitted in any relevant BMJ Author's Self Archiving Policies, I confirm this Work has not been accepted for publication elsewhere, is not being considered for publication elsewhere and does not duplicate material already published. I confirm all authors consent to publication of this Work and authorise the granting of this licence.

For peer review only

TITLE

Experiences and perspectives on chimeric antigen receptor (CAR) T-cell therapy among recipients, carers and referrers (RE-TELL): a qualitative study to inform CAR T-cell service design

Authors

Fyfe R[1,2,3], Anstis O[4], Kapadia K[4], Jordan M[4], Sword D[1], Weinkove R[1,2,5].

Affiliations

1. Malaghan Institute of Medical Research, Wellington, New Zealand

2. Te Rerenga Ora Blood & Cancer Centre, Te Whatu Ora Health New Zealand – Capital, Coast and Hutt Valley, Wellington, New Zealand

3. Faculty of Science, Victoria University of Wellington, Wellington, New Zealand

4. Health Advisory, Deloitte Limited, Auckland, New Zealand.

5. Department of Pathology and Molecular Medicine, University of Otago Wellington, Wellington, New Zealand

Corresponding author

Dr Robert Fyfe

Email: Robert.Fyfe@ccdhb.org.nz

For peer review only

Telephone: +64 4 385 5999

Address: Te Rerenga Ora Blood & Cancer Centre, Te Whatu Ora Health New Zealand – Capital, Coast and Hutt Valley, 49 Riddiford Street, Wellington, New Zealand

Word, figure and reference counts

Abstract: 300 words

Article: 5062 words

Tables: 1

References: 21

Supplemental files: 3

For peer review only

ABSTRACT

Objectives:

RE-TELL is a qualitative study which aims to understand patient, support person, clinician and co-ordinator experiences and perspectives of chimeric antigen receptor (CAR) T-cell therapy, to inform design of a clinical CAR T-cell service in Aotearoa New Zealand.

Design

Semi-structured qualitative interviews focussed on domains of: experience through treatment; elements that work well, and those that could be improved upon. Interviews used thematic analysis to identify key themes. A workshop was held to obtain participants' reflections on interim analysis and proposed improvements.

Participants:

New Zealanders with experience of CAR T-cell therapy, including recipients, support persons, clinicians and co-ordinators.

For peer review only - http://bmjopen.bmj.com/site/about/guidelines.xhtml

Results:

We interviewed 19 participants comprising: five CAR T-cell recipients, three support persons, six clinicians and five co-ordinators. 4 participants identified as Māori. Thematic analysis identified three global themes. The first, “Sociocultural factors impact CAR T access”, identified potential sources of inequity including geographic, financial and informed consent barriers. The second, “Varying emotions, roles and enablers”, identified an easier treatment experience compared to alternatives; an underwhelming cell administration process; frustration with inpatient monitoring; burden on support persons; and importance of “bridge” organisations such as charities and patient support groups. Lastly, “Golden opportunities: re-imagining CAR T service delivery”, suggested: improved geographical access to CAR T-cell therapy, while retaining consolidated clinician experience; a ‘dashboard’ with information on CAR T-cell treatment, timeframes and manufacture; a health navigator to co-ordinate non-medical aspects of treatment and signpost care; embedding of indigenous data sovereignty and ownership of cells; a cell infusion ceremony,

incorporating family involvement and Māori cultural elements; and outpatient administration and monitoring where possible.

Conclusion:

This study documented the current experience of New Zealanders receiving CAR T-cell therapy, and identified opportunities for future service development. These insights are relevant to service design within Aotearoa New Zealand, and other countries developing equitable CAR T-cell services.

Key words

Receptors, Chimeric Antigen; Immunotherapy, Adoptive; Qualitative Research; Technology, high cost; Genetic Therapy; Health Inequities; Health Services, Indigenous.

For peer review only

STRENGTHS AND LIMITATIONS OF THIS STUDY

- Strengths
  - Diverse viewpoints pertaining to CAR T-cell therapy in Aotearoa New Zealand
  - Many facets of treatment experience explored
  - Specific focus on strategies to improve indigenous Māori health outcomes
- Limitations
  - Data saturation may not have been reached for all areas
  - Further research required to develop more solutions to existing challenges

For peer review only

INTRODUCTION

Chimeric antigen receptor (CAR) T-cell therapy has become a standard of care for some relapsed and refractory B-cell malignancies [1,2]. As a personalised cell and gene therapy involving gene modification of a patient's own T-cells, access to treatment is limited by logistical challenges and cost, while varied concerns regarding the technology need to be addressed, including equity of access, optimising the patient and support person experience, and cell and data sovereignty [3,4,5]. Internationally, CAR T-cell therapy provision has not consistently ensured equity of access [6,7], and equity has been highlighted as a key area of focus for ongoing development of this treatment modality [8].

Aotearoa New Zealand does not yet have a national service for standard of care CAR T-cell delivery, with access limited to clinical trial use [9] or overseas treatment. Within Aotearoa New Zealand, cancer-specific mortality is higher for indigenous Māori than for non-Māori, including for lymphoma and myeloma [10]. As a result, there is a strong focus on designing healthcare services to rectify this inequity [11].

For peer review only

While the challenges of delivering equitable access to CAR T-cell therapy have been considered worldwide [12,13], the Aotearoa New Zealand healthcare system has unique characteristics, such as: an imperative to promote indigenous health due to the government's obligations to Te Tiriti o Waitangi/the Treaty of Waitangi (the nation's founding document, which guarantees Māori the right to access to equitable healthcare and health outcomes); low public pharmaceutical funding compared to some high income countries [14]; and wide geographic dispersal [15].

We aimed to assess the current experiences of CAR T-cell therapy for patients, support persons, clinicians and administrators within Aotearoa New Zealand, to develop insights into treatment experience, needs, and barriers to treatment; and to identify potential improvements for future services. Our overall goal was to inform design of a future national CAR T-cell service. We conducted semi-structured qualitative interviews, assessed these interviews with thematic analysis, and further refined this analysis through a co-design workshop with

For peer review only

participants, where the interim results of the thematic analysis were presented to participants for comment.

For peer review only

METHODS

For peer review only

Study design

In the RE-TELL study we recruited a total of 19 participants, including 5 patients, 3 support persons, 6 clinicians and 5 administrators, to provide a diversity of perspectives on CAR T-cell therapy. At the time of recruitment, there were an estimated 11 New Zealand patients who had received CAR T-cell therapy, given the limitations of research into a novel therapy for non-Hodgkin lymphoma. Of these, 7 were invited to participate (and accepted), wth the goal of recruiting 5 participants to provide a range of perspectives. 2 patients then became unable to participate due to health or logistical reasons. We also intended to recruit 5 support persons, clinicans and administrators, to provide additional perspectives while not prejudicing the patient voice. Participants were selected using purposive sampling to encompass a range of experiences (treated in Aotearoa New Zealand or overseas; different outcomes after treatment; different roles in a patient's care) and a diversity of backgrounds (age; ethnicity; gender; urban and rural). They were contacted by the primary investigator by e-mail with an invitation to participate and general information regarding the study.

For peer review only

A co-design methodology was chosen to take a partnership approach with patients and support persons, to understand patient experience, and to use this to develop potential improvements. Following the methodology of Boyd et al [16], the project followed the six main elements of: Engage, Plan, Explore, Develop, Decide, Change. Engage refers to establishing relationships with patients and staff to improve health services. Plan involves generating ideas about the goals of improvement work and strategies to achieve it. Explore refers to understanding patient and staff experiences, to identify areas for improvement. Develop involves turning ideas into specific improvements. Decide involves a choice between improvements and how to implement them. Change means then turning ideas into action.

For peer review only

Data collection and analysis

Utilising semi-structured interviewing techniques, we guided participants along the treatment pathway, enquiring of:treatment experience; elements that worked well; elements that could be improved on; practical needs; informational needs; emotional experiences; cultural elements; and system flow. With a deductive approach in mind, this aimed to provide a logical framework with which to structure themes and experiences, and for participants to review themes and work towards service improvements. The full interview guide is provided as Supplemental Appendix 1. Interview data were analysed using thematic analysis, with transcripts themed using the above mentioned theoretical/deductive approach. Following the framework provided by Braun and Clarke [17], the following six steps were taken to analyse the data:

1. **Familiarisation with the data:** All interviews and the focus group were transcribed and independently reviewed by RF and OA. Participants were sent their own transcripts prior to analysis, so that they were able to read through and make corrections or add clarifications. The

For peer review only

transcripts were referred to repeatedly throughout the analysis to understand and clarify meaning and resultant themes.

2. **Initial Coding.** Labels and tags were assigned within the transcripts, to identify information that related to phases of the treatment journey.
3. **Generating Initial Themes.** Coding was independently organised into practical, emotional, or cultural themes across the treatment pathway. New or novel information was highlighted to discuss between the researchers at the next phase.
4. **Reviewing and Defining themes.** RF and OA presented their coding and initial themes to each other, for agreement on global themes and subthemes.
5. **Data Charting:** Themes were mapped across the treatment pathway into multiple streams in a chart highlighting the experience of clinicians, patients and whānau.
6. **Reviewing and Refining themes.** Resultant themes were presented to participants to ratify and clarify in a focus group, and as a basis to form discussion on service improvements. These are the themes presented in this report.

For peer review only

All interviews and the workshop were facilitated by a Health Psychologist (OA) registered with the New Zealand Psychologist’s Board. Interviews were performed between 13 January 2022 and 8 April 2022. Interviews were conducted via videoconference, as COVID-19 pandemic-related restrictions precluded in-person interviews. Interviews were audio-recorded, and lasted between 28 and 60 minutes. Interviewees were given the option to review manuscripts and provide comment.

A focus group was held to review and refine themes with participants (n = 7) who self-selected to attend a videoconference workshop, in order to gain reflections on the responses and potential service improvements. During this workshop, a graphical representation of the patient journey through CAR T-cell therapy was displayed, as described by RE-TELL participants (Supplementary appendix 2). This served to summarise information gathered during the interviews, to provide logical structure to the themes by mapping them across the treatment timeline, and to help overcome health literacy barriers. The responses from workshop participants assisted with the

For peer review only

formulation of “Table 1, Global Theme 3: Future improvements to CAR T-cell pathway” and the improvements emphasised in the discussion; no new themes were identified during the workshop. The primary themes from this workshop are presented in Results – Co-Design Workshop.

Interviews were transcribed verbatim and analysed using data analysis software NVivo V.11. Transcripts were analysed and coded independently into key themes by RF and OA according to existing guidelines [17, 18]. OA had no prior involvement with the participants or CAR T-cell therapy; RF was the clinical fellow for the ENABLE trial during the study, with clinical involvement with 2 patients and 1 participant. The researchers then met to seek alignment and generate global and sub-themes collaboratively. Subthemes were allocated to provide contextual explanation of key themes, or describe less common yet important topics or emotional descriptors. Themes were formulated into a table including sample belief statements and sample quotes.

For peer review only

Statistical and ethical considerations

Ethical approval for this study was provided by Victoria University of Wellington Human Ethics Committee (ref: 29982) before any study activities. All participants gave written informed consent. The original study protocol is available as supplemental file 3.

Patient and public involvement

Patients were not involved in setting the research question or formation of the study protocol, but their responses directed the interviews, and provided the themes. We intend to disseminate the results to study participants in a newsletter suitable for a non-specialist audience.

For peer review only

RESULTS

Baseline characteristics

We enrolled 19 participants, including 5 patients, 3 support persons, 6 clinicians and 5 administrators. 4 participants were Māori, 11 New Zealand European, 2 Asian and 1 other; 12 of 19 participants (63%) were female, 7 male. The highest level of education was secondary education for 4 participants, and tertiary education for 15.

Among the 5 patients interviewed, all had B-cell non-Hodgkin lymphoma and had received CAR T-cell therapy. Their median age was 48 years (range 23-59). Three had received investigational third generation CAR T-cells within the phase 1 'ENABLE' trial in Aotearoa New Zealand [9]; one had received standard of care axicabtagene ciloleucel (axi-cel) in Australia via a government funding mechanism; and one had received lisocabtagene autoleucel in the U.S.A within a clinical trial. Four of the five patients had a complete response to CAR T-cell therapy at the time of interview.

For peer review only

Of the 3 support persons, one had supported a patient through treatment via the ENABLE trial; one through treatment in Australia; and one through treatment in the U.S.A.

The clinicians interviewed comprised four clinical haematologists who had referred patients for CAR T-cell therapy, one palliative care nurse practitioner and one research nurse. The administrators comprised a charitable services co-ordinator, a bone marrow transplant co-ordinator, a transport co-ordinator and two social workers.

Data saturation

Data saturation occurs when no additional themes emerge from the data. This was not reached in this study for all areas of analysis, and reasons for this are explored in the Discussion. Specifically, many of the subthemes mentioned in Global Theme 3 did not reach saturation, as there were a multitude of potential areas improvements raised, which were not discussed with all participants.

For peer review only

Interviews: Key domains and themes:

Themes arising from the interviews were organized into three global themes, before subclassification into 8 domains and 23 subthemes (see Table 1). The first global theme, Sociocultural factors impact CAR T access, relates to both the challenges that patients and clinicians face in attempting to access CAR T-cell therapy, and the barriers and inequities that may prevent others from accessing the treatment. The second global theme, Varying emotions, roles and enablers, describes the experience of treatment within New Zealand or overseas, including the ancillary services in place to support patients. The final global theme, Golden opportunities: re-imagining CAR T service delivery, explores the ways that a future national CAR T-cell therapy service might improve treatment delivery.

For peer review only

1

Table 1: Global themes, key domains, subthemes and sample quotes		
Relevant domains	Subtheme	Sample quotes
Global theme 1: Sociocultural factors impact CAR T access		
Equity	Potential inequity stemming from lack of access	"To be equitable, you need to make sure it is accessible to everyone, and we can't just rely on clinical trials and compassionate access programmes" [C1].
	Geographic barriers	"The first barrier is distance" [C1]<br>"I understand you can't have CAR T in all centres. I just think one is probably not enough for many patients" [C1]<br>"You are asking someone who has a limited support network to fly… it's hard for the patient psychologically"[C1]
	Informed consent	"As soon as you start talking about how risky things are – a Māori worldview is often quality over quantity…. In a medicolegal worldview, we go into the nth degree about every small little potential risk, such that [Māori] will start to get scared and go well, actually, I don't want this." [C5]<br>"You should give me information that I can just hand to my mother and she understands it" [P1]
	Financial barriers	"People just don't have enough money… for financial support, transitioning from being a full time employee to having to stop." [A4]<br>"We say to patients we have this intensive treatment – it's fully funded, all public… but we have no mechanism to help you stay down here, and you've got to a charity [for accommodation]" [A2]
	Awareness of trial	"At least a couple of patients initiated the conversation about wanting to be part of the CAR T-cell trial" [C4]<br>"People don't know where to look sometimes" [C6]
	Requirement for advocacy	"Our health system requires you to navigate it yourself, it's so patient unfriendly" [P1]

For peer review only

**Global theme 2: Varying emotions, roles and enablers**		
Patient experience	Underwhelming administration of cells	"It's just a shot in your arm and then you're done… anti-climactic". [P5]
	Treatment experience easier compared to chemotherapy	"It was quite chill… chemo's a hell of a lot worse" [P5]<br>"A walk in the park" [P2]<br>"It was actually the first time through the whole cancer journey that I got to sleep a lot… I just rested" [P3]
	Frustration with inpatient monitoring	"I was so bored I went out to strip the bed, got some new sheets and made my bed up" [P2]
Support person experience	Emotional burden	"The relief to get him to the hospital and know that somebody else was looking after him, and it wasn't me that was having to keep things going every day" [S3]<br>"There's no care for the carers" [S1]
	Requirement to provide care for patients	"I felt like I'd gone from being a wife to a carer" [S3]
	Role in advocacy for patients	"Half the time I don't think [the patient] knew what day it was" [S3]
Bridges	Charities	"Sometimes patients and families do like to talk to an organization that understands, but is separate to the hospital… so they won't be judged" [A1]<br>"There is a period of time there where the hospital finishes their treating, and they may not be so unwell that a hospice is involved, but they're in that middle zone and it's really stressful." [A1]
	National Travel Assistance programme	"It's not meant to cover [all travel costs], but it's meant to … eliminate the barrier of those upfront costs" [A5]
	Patient support groups	"Show respect for what they've gone through and being able to articulate that experience to others" [A1]
**Global theme 3: Golden Opportunities: re-imagining CAR T service delivery**		

For peer review only

Centralised point of information	Accessible prepared resources	"It's good to have it delivered through a video. It's a bit easier to comprehend" [P1]<br>"Knowledge is power… if you've got the information and know what to expect, it takes a lot of the fear out of the process" [P3]
	Health navigator	"Someone who's thinking about the whole patient's life and experience going through the process, not just the medical side of it… a generalist, and then the specialists are doing their work". [P1]
Alterations to treatment delivery	Outpatient therapy	"I could have done a lot of that as an outpatient" [P2]
	Ceremony for cell infusion	Having family members, and potentially referring clinicians present for cell infusion: "It will show [patients] how important it is" [C5]
	Need for appropriate accommodation	"Really inappropriate [to require family members to sleep on the floor]" [P3]
Service design	Clinical staff experience	"You need to have [dedicated] staff because it's a unique therapy… [bone marrow transplant co-ordinators] couldn't just absorb [a CAR T-cell therapy role], they'd need extra resource". [A2]
Indigenous rights	Data sovereignty	"Is it information for information's sake, or is there a purpose at the end?" [C5]
	Cell sovereignty	"They told us what they were going to do with the stem cells if they didn't need them.. whereas in Australia? We never got those options". [S2]<br>"You almost want a courier tracking system [for cells]" [C5]

1

2 Global theme 1: Accessing CAR T-cell therapy

For peer review only

Potential inequity stemming from lack of access: Current mechanisms of access to CAR T-cell therapy in Aotearoa New Zealand are via clinical trial, self-funded treatment overseas, or government funded treatment overseas. There was broad agreement among those interviewed that current access to CAR T-cell therapy is inequitable, with the impression that those able to access treatment are likely to be more health literate, have more financial resources, are more likely to reside in an urban centre, and exist within societal structures that enable their health literacy and ability to navigate the health system.

Requirement for self-advocacy: Noting the barriers to accessing CAR T-cell therapy identified above, a frequently raised theme was that patients and support persons were required to advocate for themselves, either to access CAR T-cell therapy in the first instance, or to shape their clinical care to fit their needs. Although referrals for CAR T-cell therapy occurred via a patient's haematologist/oncologist, several patients had to raise the option of CAR T-cell therapy with their treating clinician before this was

For peer review only

considered. Many felt that the in the absence of standard-of-care CAR T-cell therapy in Aotearoa New Zealand, knowledge of referral pathways relies on ‘word-of-mouth’ communication.

Geographic barriers: Aotearoa New Zealand is a country with a low population density, long transport times between centres, and is distant from international centres that offer CAR T-cell therapies. Geographic inequity was identified as a factor impacting patients’ ability to access therapy. Geographic barriers impacted people’s finances and ability to care for their children, and resulted in time away from support networks and whānau (family), and concerns about housing and job security.

Informed consent: It was noted that many aspects of accessing treatments in Aotearoa New Zealand require an ability to navigate a complex medical system. In particular, the current process of consent for investigational treatments requires extensive cataloguing of possible risks of therapy. Participants commented that if this is provided without an equivalent

For peer review only

explanation of potential benefits, patients may leave with the understanding that CAR T-cell therapies are riskier than they truly are (rather than accepting that the Western medicolegal paradigm requires robust documentation of each risk) [see: Table 1; global theme 2]. While this issue with informed consent was noted by both NZ European and Māori patients, it was thought that it may have greater impact on Māori patients. Interviewees noted that this could be further exacerbated by differences in health literacy and an inability for many clinicians to provide information within a Te Ao Māori (Māori world) framework, which could contribute to an erosion of trust in the health system.

Financial barriers: Although patients attempting to access CAR T via clinical trial in Aotearoa New Zealand experienced barriers, these were even more formidable for patients attempting to self-fund CAR T-cell therapy overseas. Among those accessing overseas treatment, patients and family were required to identify overseas hospitals with actively enrolling CAR T-cell therapy programmes; liaise with these hospitals and arrange transfer of medical information; fundraise high sums through combinations of personal

For peer review only

funds, fundraising events or crowdfunding; and co-ordinate logistics to arrive at the treating hospital – all while unwell with their disease. Unsurprisingly, it was noted that many who considered exploring treatment via this route were eventually unable to access this.

Global theme 2: Varying emotions, roles and enablers

Treatment experience easier than chemotherapy: The predominant theme regarding the treatment experience was the relative tolerability of CAR T-cell therapy. New Zealanders who accessed CAR T-cells within the ENABLE trial did so as a last-line therapy, so had experience of numerous chemotherapy cycles, and often of stem cell transplantion. Participants reported that, although there were adverse effects, CAR T-cell therapy was much more comfortable and tolerable than their prior treatments. Indeed, the primary difficulty with CAR T-cell therapy was reported to be the prolonged inpatient monitoring (typically 14 days), or (for the recipient of axi-cel in Australia) monitoring in an Intensive Care Unit for cytokine release syndrome (CRS), while feeling well. Additionally, many of the treatment challenges highlighted

For peer review only

were not primarily attributable to physical adverse events, but rather to socioeconomic factors. For those treated in Aotearoa New Zealand, these included: accessing suitable accommodation for patient and support person; affording groceries; and compensating for loss of income while a support person relocated to accompany the patient. For those treated outside Aotearoa New Zealand, these included: identifying overseas treating centres and securing a treatment place; complex and expensive logistical planning; and balancing having a support person at the treating centre to support the patient with maintaining family life at home. It was commented that conducting follow-up in an outpatient setting would have overcome many of the challenges of treatment (see Global Theme 3).

Underwhelming administration of cells: It was noted that the infusion of CAR T-cells could be anti-climactic, since it involved a very brief cell infusion. While some patients found the moment of CAR T-cell administration momentous and significant, others felt it was underwhelming, and detracted from the treatment experience.

For peer review only

Support person experience: Our interviews identified the crucial role that support persons played in the care of patients receiving CAR T-cells. This included assistance with monitoring for CAR T-cell related adverse events, and co-ordinating the many non-medical challenges that arise as a patient goes through cancer treatment. In addition, support persons often played an active role by advocating for the patient. Reckoning with these new responsibilities, while managing the psychological stress of a close family member's life-threatening illness, presented a significant emotional burden for support persons.

Bridges: A characteristic of CAR T-cell therapy treatment for New Zealanders was the need for external organisations to serve as "bridges" to cover gaps in patient care that currently exist in the funded health system. These "bridges" include charitable organisations, such as Leukaemia and Blood Cancer NZ, who provide financial assistance for travel and accommodation costs during treatment, psychosocial support, and participation in support groups for

For peer review only

patients to meet other patients receiving similar treatment. Such support groups also arise organically, such as via social media. Community hospices also provide a valuable service providing both end-of-life care and community outreach palliative care input.

The current healthcare system provides a National Travel Assistance programme, which covers a portion of the logistical and accommodation costs for patients and support persons travelling out-of-region for medical treatment. However, the gaps in coverage of this programme result in costs falling to either patients or to charitable organisations.

Global theme 3: Golden opportunities: re-imagining CAR T service delivery

The final major theme related to improvement opportunities for a future national CAR T-cell therapy service.

Centralised point of information: It was recognized that the introduction of a CAR T-cell therapy should be accompanied by education and support

For peer review only

structures to help clinicians and patients unfamiliar with the therapy to access it. For clinicians, this would require visible and accessible specialists to liaise with, and clear and accessible guidance of the referral process. For patients, this involves quality education materials to explain the treatment in understandable terms, preferably in video form in addition to text.

Health navigator: It was identified that a health navigator (sometimes referred to internationally as a “concierge”) could serve a role as a patient liaison throughout treatment, coordinating both the medical and non-medical aspects of care (including logistic, financial and psychosocial considerations), providing patient advocacy, and education about aspects of treatment. An idiosyncratic element of CAR T-cell therapy is the cell manufacturing period, leading to a period of uncertainty for patients and referring clinicians as the outcome of CAR T-cell manufacture is awaited. Both clinicians and patients identified a potential role for a ‘cell tracker’, with which the progress of cell manufacture could be tracked over time, to manage expectations and provide reassurance.

For peer review only

Changes to treatment delivery: Several potential alterations to current CAR T-cell therapy clinical delivery were identified. First was the possibility of conducting a greater proportion (or even the entirety) of monitoring as an outpatient following cell infusion. This was felt to offer benefits for patient wellbeing as well as for health resourcing. Secondly, to address the underwhelming nature of cell administration, a ceremony for CAR T-cell administration was suggested, to imbue the procedure with a reverence befitting the occasion. This could involve family, friends or referring clinicians, either in person or via videoconference, as well as additional cultural or religious elements such as presence of kaumatua (Māori elders) and a karakia (blessing).

Dedicated clinical staff: It was identified that a CAR T-cell therapy service requires dedicated resourcing and training, rather than unfunded incorporation into overstretched bone marrow transplantation services. Treatment hubs

For peer review only

should have dedicated CAR T-cell staff to allow a consolidation of skills and experience, and to ensure safe treatment delivery.

Indigenous sovereignty: Finally, it was identified that a future CAR T-cell service should include provisions to respect indigenous sovereignty over data and cells. For data, this should include anonymizing or de-identification of data shared beyond treating clinicians (unless explicit consent is given), as well as the understanding that data would lead to improvements for the patient group that the data is sourced from. For cells, this would involve explanation of where cells would be located, what processes would be undertaken with them, and what would happen to remaining cells that are not required for their cellular therapy. As an example, one Māori participant underwent a cell collection procedure in Aotearoa New Zealand, before undergoing cell collection for a commercial CAR T-cell therapy overseas. They highlighted that in Aotearoa New Zealand they were informed about location of their cells during manufacture, and were given the option to either donate or dispose of cells that were not required for treatment. Conversely,

For peer review only

during their treatment overseas they were not informed of the location of their cells or the manufacturing progress, nor given options about fate of their cells.

For peer review only

Co-design workshop

Participants noted that, for patients and support persons attempting to access CAR T-cell therapy, many of the challenges to be navigated are at or before the start of the pathway, and suggested refinements to the pre-referral process, and monitoring of referrals to ensure access is equitable.

Participants commented that, although many elements of the current pathway could be improved upon, including consent processes, support services and cultural elements, the primary focus of patients and support persons is to access CAR T-cell therapy, and that the need to improve these areas should not prevent initial adoption of the therapy.

Participants were asked to comment on which of the potential improvements (broadly identified in Global Theme 3) they would prioritise. This resulted in strong support for the health navigator role (to assist patients and families with non-medical and medical navigation of the health service and the effect that cancer treatment would have on their lives); the need for culturally appropriate

For peer review only

care for all patients; and a centralised dashboard for information (to provide information about how to access CAR T-cell therapy and what treatment would entail). Elements such as a tracker for cells after extraction, and a ceremony around cell infusion, were considered improvements but less crucial to the success of a CAR T-cell therapy programme.

DISCUSSION

Aotearoa New Zealand has an unmet need for funded CAR T-cell therapies for B-cell non-Hodgkin lymphomas and other B-cell malignancies. As we expect CAR T-cell therapy to become a standard of care in the future, it is important that consideration is given to the perspectives of patients, support persons, clinicians and administrators, in design of a national service. Therefore, we performed this qualitative study to generate insights into the current experiences of CAR T-cell therapy, and the areas which could be improved upon.

For peer review only

Elements of the current service were praised by participants. First, CAR T-cell therapy was considered more tolerable than other treatments such as chemotherapy and autologous stem cell transplantation. Second, the role of charities and other organisations in expanding on the support provided by healthcare providers, and mitigating the challenges facing patients and support persons through treatment was applauded. Third, the use of strategies to respect tissue sovereignty within the ENABLE trial was thought to lay important groundwork for further research in this area in future.

We identified several areas for potential improvement of CAR T-cell delivery. First were strategies to improve communication with patients and referring clinicians, and therefore minimise some of the barriers to access identified in Table 1. Some patients had to ask to be referred for the CAR T-cell clinical trial, suggesting that a more robust system of informing clinicians of open clinical trials is required. For a standard of care service, strategies could include a centralised ‘dashboard’ for new CAR T referrals (with clear guidance for clinicians on eligibility, process and timeframes), and high-quality educational

For peer review only

materials for patients and family in understandable language. Communication could also be improved with a ‘tracker’ of cell collection and manufacture, which would provide patients and referring clinicians with an insight into timeframes.

Other improvements to improve the treatment experience included a transition to outpatient management, which would result in benefits for patients (improved psychosocial wellbeing and avoidance of infection while in hospital), alongside resourcing benefits (reduced inpatient burden, and less overall staff input). Such a transition would require more robust assistance from a National Travel Assistance programme, to ensure that treatment remains a realistic option for patients with fewer resources. A ceremony for CAR T-cell administration, including involvement of family and referring clinicians, and incorporating cultural or religious elements, may be appreciated by many patients.

Given the ethnic health disparities in Aotearoa New Zealand, including poorer cancer-specific survival for non-Hodgkin lymphoma and myeloma among Māori [10], a national CAR T-cell service will need to be designed with an explicit

For peer review only

equity focus to ensure it improves Māori health outcomes and avoids replicating pre-existing issues with access to treatment and informed consent. Given the socioeconomic disparities since colonisation, this may require increased support to access treatment and to mitigate financial consequences for the patient and support persons relocating for treatment. It would also require culturally appropriate care within the CAR T-cell therapy team. Patient autonomy over data and cells was strongly valued, with the option to decide on how both are used.

Finally, we identified the need for treating hubs with consolidated experience and skills, to ensure that clinicians can deliver this new therapy safely. However, it was felt that keeping CAR T-cell therapy in a single treating centre, even in a country with a relatively small population such as Aotearoa New Zealand, could perpetuate existing geographic inequities. Therefore, consideration will need to be given in the medium term to balancing the relative trade-offs between efficiencies gained from consolidated care and the benefits to patients of care closer to home.

For peer review only

One of the key strengths of this study is the diverse viewpoints obtained by enrolled participants, providing a "360 degree" view of the CAR T-cell therapy process in Aotearoa New Zealand – that is, not only patients, but support persons, clinicians and administrators. We intentionally enrolled patients and support persons with experience of both positive and negative outcomes after CAR T-cell therapy, as well as participants with a range of ages and ethnic backgrounds. This diversity of viewpoint, we feel, improved our ability to obtain a broad range of data on current experience, and helped us to identify new solutions.

Another strength of our study is the wide range of topics covered relating to CAR T-cell therapy. The flexibility of semi-structured interviews, as well as the wide variety of associations of participants to CAR T-cell therapy, means that this project considered many different facets of the treatment pathway, from the individual (e.g. patient and support person experience and communication between clinicians) to the system-level (identifying areas of inequity, and how

For peer review only

a future service should be designed). This data-driven approach, as opposed to a theory-driven approach, meant that the insights from participants could extend beyond the areas foreseen by the prespecified interview template.

This study was designed in discussion with Māori, and specifically aimed to recruit Māori participants, to inform ways a CAR T-cell service should be designed to improve Māori health outcomes. Our findings highlight the need for further research regarding tikanga, or Māori custom, relating to CAR T-cell therapies, including the role and form of karakia (blessings) at the time of cell collection, administration and disposal. Our findings regarding the needs of support persons touch on the importance of a holistic approach to health outcome evaluation, which for Māori includes mental, spiritual and family/community wellbeing, as well as physical health (the Te Whare Tapa Whā model [19]). Such work should be conducted using a kaupapa Māori (Māori-led) approach, to ensure the design of a new health pathway actively addresses inequities in Indigenous health outcomes.

For peer review only

A limitation of this study is that by interviewing a wide group of participants, and having a semi-structured interview with scope to follow the interviewee to cover topics in more depth, data saturation was not reached in all areas. Indeed, given the limited numbers of New Zealanders who have received CAR T-cell therapy, data saturation may not have been possible. A further restriction to research into patient experience of relapsed/refractory non-Hodgkin lymphoma is that the experience of patients not responding to treatment may be lost due to rapid progression of their disease. While we attempted to ameliorate this by interviewing support persons, clinicians and administrators, this may have served to dilute the patient voice. We attempted to mitigate this by testing the thematic analysis within a participant workshop, to gain reflections on themes raised by others. We also note that the themes we have identified, including barriers to accessing CAR T-cell therapy, align with those identified elsewhere [3,6,8,13,20]. We also acknowledge that applying a deductive approach to thematic analysis may have restricted the reporting of themes amongst participants. Guiding participants along the treatment pathway by way of discussion was a strategy to assist in reducing distress (patients and whanau

could anticipate the next step in questioning), and also to assist in the building of a re-imagined pathway. An alternative approach could have been to use an inductive approach, with a broader approach to questioning. This may have resulted in themes that did not necessarily emerge in this work. In fact, during the feedback hui, participants commented that the journey before seeking treatment, and after finishing treatment, was of equal importance to them; however, this was beyond the scope of this project.

The focus of the interviews in this study was on the treatment experience and on areas of focus for future service design. While specific suggestions arose that might be incorporated into a future pathway (see Table 1, Global Theme 3), these suggestions alone are not sufficient to address all of the challenges that were identified, particularly with regards to equity. It is hoped that this work will help to identify areas for future focus; such research could include a more comprehensive identification of strategies to address the challenges raised.

For peer review only

While other studies have assessed the patient experience of CAR T-cell therapy via qualitative interviews [13,21], or assessed the principles required for a CAR T-cell service [8], our study is the first to examine this in Aotearoa New Zealand. This study emphasises the importance of co-design to elevate the voice of the indigenous Māori population, and to address the current severely limited access to CAR T-cell therapy. The suggestions unearthed in this study should help development of an intentionally designed service that results in health gains for Māori, and does not exacerbate health inequities.

In summary, the RE-TELL study has defined the current experience of New Zealanders receiving CAR T-cell therapy; identified some of the barriers to those seeking treatment; and identified improvements for a future service, as well as areas for attention for those designing this service. Although our research is specific to Aotearoa New Zealand, elements of our findings are likely to apply to other countries intending to develop CAR T-cell therapy services, particularly those countries in which indigenous people experience health inequities.

For peer review only

ACKNOWLEDGEMENTS

The authors would like to thank the participants for their time and insights, and for their willingness to share their experiences. We acknowledge those who have passed, and uphold this study as part of their legacy. We also acknowledge Myra Ruka for input into design of the study and review of the manuscript. This study was presented in a provisional form at the Blood Conference 2022.

FUNDING

This study was supported by funding from Janssen-Cilag Pty Ltd to Deloitte and the Malaghan Institute (grant: N/A). RW is supported by a Clinical Practitioner Fellowship from the Health Research Council (19/139). RF is supported by the LifeBlood Trust. The Malaghan Institute acknowledges philanthropic funding support from David Downs and The Thompson Family Foundation.

COMPETING INTERESTS

For peer review only

RF, RW and DS are employees of the Malaghan Institute of Medical Research, which received funding from Janssen-Cilag Pty Ltd towards the cost of this research. OA, KK and MJ are employees of Deloitte, which received funding from Janssen-Cilag Pty Ltd through a contract for services. Janssen-Cilag had no involvement in the conduct of the research, data collection, data analysis, or in preparation or approval of the manuscript.

CONTRIBUTORSHIP STATEMENT

RF, OA and RW designed the study and wrote the study protocol. RF, OA, KK and MJ performed study procedures. RF, OA, DS and RW analysed the data and drafted the manuscript. All authors reviewed and approved the final manuscript.

DATA SHARING STATEMENT

Given the commitments made to participants regarding deidentification of data shared from this research, the authors are not able to share participant transcripts from interviews.

For peer review only

ETHICS APPROVAL STATEMENT

Ethical approval for this study was provided by Victoria University of Wellington Human Ethics Committee (ref: 29982) before any study activities were commenced. All participants gave written informed consent.

For peer review only

REFERENCES

1. Locke F, Miklos D, Jacobson C et al. Axicabtagene Ciloleucel as Second-Line Therapy for Large B-Cell Lymphoma. *N Engl J Med* 2022; 386: 640-654
2. Schuster S, Bishop M, Tam C et al. Tisagenlecleucel in Adult Relapsed or Refractory Diffuse Large B-Cell Lymphoma. *N Engl J Med* 2019; 380:45-56.
3. Jommi C, Bramanti S, Pani M et al. CAR T-Cell Therapies in Italy: Patient Access Barriers and Recommendations for Health System Solutions. *Front. Pharmacol* 2022; 13:915342.
4. Lim F, Yunxin C, Huang FJ et al. A unique hub-and-spoke model to optimize patient management in lymphoma using novel CAR T-cell therapy in southeast and south Asia. *Haematol Oncol* 2022; doi: 10.1002/hon.3057. Online ahead of print.
5. Hudson M, Mead A, Chagné D et al. Indigenous Perspectives and Gene Editing in Aotearoa New Zealand. *Front Bioeng. Biotechnol* 2019; 7: 70.
6. Kamal-Bahl, Puckett J, Bagchi I et al. Barriers and solutions to improve access for chimeric antigen receptor therapies. *Immunotherapy* 2022; doi: 10.2217/imt-2022-0037. Online ahead of print.
7. Emole J, Lawal O, Lupak O et al. Demographic differences among patients treated with chimeric antigen receptor T-cell therapy in the United States. *Cancer Medicine* 2022; doi: 10.1002/cam4.4797. Online ahead of print.
8. Gagelmann N, Sureda A, Montonto S et al. Access to and affordability of CAR T-cell therapy in multiple myeloma: an EBMT position paper. *Lancet Haemtol* 2022; 9:e786-95.
9. George P, Dasyam N, Giunti G et al. Third-generation anti-CD19 chimeric antigen receptor T-cells incorporating a TLR2 domain for relapsed or refractory B-cell lymphoma: a phase I clinical trial protocol (ENABLE). *BMJ Open* 2020; 10(2):e034629.
10. Gurney J, Stanley J, McLeod M et al. Disparities in cancer-specific survival between Māori and non-Māori New Zealanders, 2007-2016. *JCO Glob Oncol* 2020; 6:766-774.

For peer review only - http://bmjopen.bmj.com/site/about/guidelines.xhtml

11. Ministry of Health. New Zealand Cancer Action Plan 2019-2029 – Te Mahere mō te mate Pukupuku o Aotearoa 2019-2029. *Ministry of Health* 2020.

12. Zozaya N, Villaseca J, Abdalla F et al. A strategic reflection for the management and implementation of CAR-T therapy in Spain: an expert consensus paper. *Clin Transl Oncol* 2022; 24(6):968-980.

13. Castillo G, Lalu M, Asad S et al. Navigating choice in the face of uncertainty: using a theory informed qualitative approach to identifying potential patient barriers and enablers to participating in an early phase chimeric antigen receptor T (CAR-T) cell therapy trial. *BMJ Open*. 2021; 11(3):e043929.

14. Chow R, Bradley E, Gross C. Comparison of Cancer-Related Spending and Mortality Rates in the US vs. 21 High-Income Countries. *JAMA Health Forum*. 2022; 3(5):e221229.

15. Weinkove R, George P, Ruka M et al. Chimeric antigen receptor T-cells in New Zealand: challenges and opportunities. *N Z Med J* 2021; 134(1542):96-108.

16. Boyd H, McKernon S, Mullin B et al. Improving healthcare through the use of co-design. *N Z Med J* 2012; 125(1357):76-87.

17. Braun V, Clarke V. Using thematic analysis in psychology. *Qualitative Research in Psychology* 2006; 3(2):77-101.

18. Fugard A, Potts H. Thematic analysis. *SAGE Research Methods Foundation* 2019; https://dx.doi.org/9781529748659.

19. Durie M. Whaiora: Maori Health Development. *Auckland: Oxford University Press* 1998; pp68-74.

20. Buradagunta C, Garacci Z, D'Souza A et al. Socioeconomic disadvantage contributes to ethnic disparities in multiple myeloma survival: a matched cohort study. *Blood Cancer Journal* 2022; 12:82.

21. Shah N, Delforge M, San-Miguel J et al. Patient experience before and after treatment with idecabtagene vicleucel (ide-cel, bb2121): qualitative analysis of patient interviews in the KarMMa trial. *Leuk Res* 2022; 120:106921.

Supplementary file 1: Semistructured interview guide

For patients and support persons

Introduction and explanation of session

Entry questions

- Age
- Ethnicity
- Education
- Whether received treatment or supported someone through treatment

Experiences through treatment

- Describe your CAR T-cell treatment
  - When did you first hear about CAR T-cells?
  - How did you travel to obtain CAR T-cells?
  - How did you find the treatment process?
    - How long did you spend in another city obtaining CAR T-cell treatment?
  - How did you find returning to your home city after treatment?
    - How well did your different health professionals co-ordinate together?
    - How did you find the follow-up period after having treatment with CAR T-cells?
- What was the emotional impact of the treatment?
  - Were your expectations met by the treatment?
  - Is there anything that could have made the treatment easier for you?
- What was the impact of your treatment on your support person and wider whānau/family?
- What was the financial impact of your treatment?
  - What support was provided during this time?
  - What other supports could have helped?

Treatment aspects that worked well

- What parts of your treatment journey do you think worked well?
- What parts of your treatment should be preserved, or kept, if more people were treated with CAR T-cells in the future?

Treatment aspects that could be improved upon

- What parts of your treatment do you think could be improved upon?

- How could the process of travelling to-and-from the CAR T-cell treating centre be improved?
- What parts of your medical treatment did you find most challenging?
- Were the explanations about CAR T-cells accurate for your experience?
  - If not, why not?

For peer review only

- What else would you have wanted to know about before receiving CAR T-cell therapy?

- Were there any emotional supports that could have made this process easier for you?
- What challenges did you face in accessing CAR T-cells that you think could be avoided in future?
- Did you feel your culture was respected throughout your treatment?
    - If not, why not?
- Did you feel your religion was respected throughout your treatment?
    - If not, why not?
- What challenges did you face in accessing CAR T-cell treatment?
    - How could this be addressed in future?

For peer review only

For clinicians and administrators

Introduction and explanation of session
Entry questions

- Age
- Ethnicity
- Education
- Role in CAR T-cell treatment
- Experience with CAR T-cell treatment

System flow

- How well does the current healthcare system facilitate access to CAR T-cell therapy?
- How efficient do you think the current system is?
- What are the current barriers to patients accessing CAR T-cell treatment?
- What are the current barriers to patients moving through CAR T-cell treatment?
- What factors help patients access CAR T-cell treatment?
- Do you think current access to CAR T-cell therapy is equitable?
    - If not, what are the main current barriers?
        - How could these be addressed?

Staff experience of the CAR T-cell therapy pathway

- Did you have all the information you needed to help your patient access CAR T-cell treatment?
    - What else would you have wanted to know prior to the treatment?
- What resources could help improve the CAR T-cell treatment pathway from a practical perspective?
- What was your experience of the CAR T-cell treatment programme from an emotional perspective?
    - Are support resources needed for staff during this treatment?

For peer review only

Supplementary file 2: Visual representation of patient journey through CAR T-cell therapy

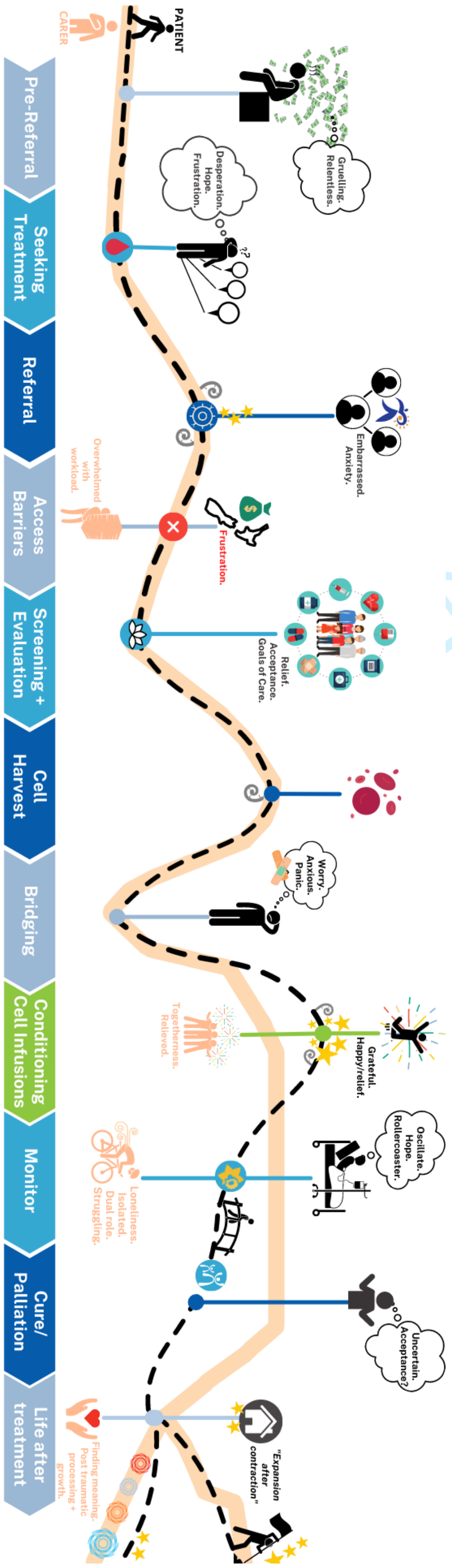

Supplemental file 3: Original RE-TELL study protocol

Full study title: REviewing patients and carers' experiences to inform the co-design of an optimal CAR T-cELL therapy service (RE-TELL study)

Protocol version: 1.0

Protocol date: 17 December 2021

Principal investigator: Robert Fyfe

Co-investigator:
Robert Weinkove
Olivia Anstis
Stephanie Easthope
Kushant Kapadia

For peer review only

1. Background and significance

Chimaeric antigen receptor (CAR) T-cell therapy is an emerging cellular therapy for the treatment of cancer. It involves using a patient's own T cells and modifying them with a CAR to both specifically target their cancer and then upregulate their immune system to eradicate the cancer. At this stage it is used primarily for blood cancers such as non-Hodgkin's lymphoma or B-acute lymphoblastic leukaemia, but the technique is being used to develop CAR T-cells to treat both other blood cancers such as myeloma as well as potentially solid cancers. This technique may have increasing use in the future to both use a new form of immunotherapy to treat cancers that do not respond to conventional chemotherapy, and also are in most cases more tolerable to patients than the untargeted approach of chemotherapy.

Current access to CAR T-cell therapy in New Zealand is limited to either: (a) government funding for standard-of-care CAR T-cells in exceptional circumstances, (b) private funding, or (c) through the ENABLE clinical trial, run by the Malaghan Institute of Medical Research. As access to this treatment is likely to improve in future, there is currently an opportunity to use the experience of New Zealanders who have received CAR T-cells to design how an optimal CAR T-cell care pathway rather than instituting a service in an ad-hoc fashion once funding is approved.

There are unique aspects to the clinical delivery of CAR T-cells, including the cell harvest, cell manufacture, and monitoring for toxicities and infection after cell delivery, which are different to other current treatments. There are also distinctive aspects to the New Zealand context, including the role of Māori engagement and kaitiakitanga (guardianship) in shaping new treatments , the equity focus, and a unique regulatory environment (Weinkove, 2021). Therefore, a treatment pathway tailor-made for New Zealand is needed, rather than translocating other system imprinting them on a New Zealand context.

Our aspiration is that the CAR T-cell treatment pathway designed through this work can then inform the design of CAR T-cell treatments delivered in New Zealand in the future.

2. Study hypothesis and aims

The hypothesis of this study is that the current treatment pathway for CAR T-cell therapy can be improved upon for future treatments by the involvement of both patients, support persons, clinicians and administrators in a co-design project.

For peer review only - http://bmjopen.bmj.com/site/about/guidelines.xhtml

The aim is to, by first interviewing stakeholders and then having focus groups for group discussion, produce an optimal treatment pathway for future CAR T-cell therapy to use.

3. Administrative organisation

Francis Health is an internationally networked health and disability consultancy firm, delivering strategic planning, service design, service improvement, research and evaluation across New Zealand, Australia and the United Kingdom.

Francis Health has operated for 19 years with a track record of successfully supporting healthcare organisations in New Zealand. The Francis Health NZ team has 15 staff with a range of expertise, both clinical and non-clinical. We have a large network of expert associates available to support programmes as needed.

4. Study design

Study population

At this point in time there are a very small number of adults in New Zealand who have undergone CAR T-cell therapy. The RE-TELL study team are aware of 11 adults, with there being potentially several others currently unbeknownst to the study team.

The inclusion criteria for patients and support persons are:
- Have undergone, or have supported someone with CAR-T therapy
- Aged 16-80 years
- Have access to a telephone or internet for the purposes of an interview
- Able to provide informed consent to participate in the study in accordance with ICH-GCP (International Conference on Harmonisation, Good Clinical Practice) principles

In addition, we will recruit five clinicians (including doctors, nurses and administrators) for interviews, and four administrators (including NGO providers, treatment co-ordinators, ward clerks and members of charitable trusts).

Inclusion criteria will be:
- Have been involved in the treatment of someone with CAR T-cell therapy in New Zealand
- Have access to a telephone or internet for the purposes of an interview
- Able to provide informed consent to participate in the study in accordance with ICH-GCP principles.

Sample size determination

For peer review only

The justification of sample size in the current qualitative study is based on the context of the research, and the availability of participants. It is understood that in qualitative research, narratives from as few as one participant can provide value (Boddy, 2016), and there is no set standard in terms of number of participants required to reach thematic saturation (Sandelowski, 1995). The context of this study is limited to the experience of patients who have undergone CAR-T therapy in New Zealand, and their carers. Currently only 11 patients have undergone CAR-T therapy in New Zealand. Therefore this study aims to recruit up to six patients and four support persons. In addition, we will aim to recruit five clinicians (Specialists, Registrars, Specialist Nurses (including Leukapheresis Nurse), Ward Nurse) and four healthcare administrators (NGO providers, treatment coordinators and ward clerks). This gives a total of 19 participants in the study.

5. Study procedures

Participant selection

We will use purposive sampling in order to enrol participants with a breadth of experience (treated in NZ vs. overseas; different cancers; different outcomes) and also breadth of backgrounds (age, ethnicity, gender, rural vs. urban). The intention will be to recruit in line with population parity with regards to age, ethnicity and gender; however given the small numbers of eligible participants and possible challenges with recruitment, it is not felt feasible to undertake representative sampling.

In addition, we will recruit five clinicians (including doctors, nurses and administrators) for interviews, and four administrators (including NGO providers, treatment co-ordinators, ward clerks and members of charitable trusts). Again, purposive sampling will be used to identify a breadth of experience and perspectives.

Recruitment

Patients and support persons will be recruited through several channels. Firstly, those who have undergone CAR T-cell therapy through the ENABLE clinical trial, who are to date the only patients to have received CAR T-cells in New Zealand, will be contacted and invited to participate. In addition, patient advocates and Leukaemia Blood and Cancer NZ will be contacted to identify patients and their

support persons who have received CAR T-cell therapy overseas, either via self-funding or government funding.

Clinicians and administrators will be approached by the researchers on the basis of the relevance of their role to the CAR-T care pathway. Each of these participants should play an active role in the prescription, administration and/or coordination of CAR-T therapy in New Zealand. This would include:

- Prescribing clinicians
- Referring clinicians
- Nursing staff (e.g. Nurse Specialist, Leukapheresis Nurse, Ward Nurse)
- Treatment/Study Coordinators
- NGO providers (e.g. Leukaemia and Blood Cancer Foundation)
- Administrative staff
- Ward clerk

Participant screening

Prospective participants will be initially contacted by the primary investigator to discuss possible interest in the study, and to provide general information including the participant information and consent form.

Given that the patient population may be vulnerable, having lived with a cancer diagnosis and undergone treatment as part of a clinical trial, follow-up contact following indication of interest to participate will be completed by a Health Psychologist registered with the New Zealand Psychologists' Board. Health Psychologists adhere to a strict code of ethics, which ensures that vulnerable persons are not coerced into participating in research. In addition, the psychologist will be able to assess whether the person is of sound mind to participate. Criteria would include any observable difficulties with memory, concentration, coherence, orientation, speech and attention. It is expected that there may be some limitations given the effects of treatment and the age of participants, and this will be taken into consideration during the screening process. Health psychologists are also attuned to changes in affect that may indicate

For peer review only

participant distress. If a participant is noted to be suffering undue distress as a result of participating, they will be asked whether or not they would like to continue.

Consent

Prospective participants will be contacted via phone or via e-mail. After a discussion of the project, a patient information and consent form will be either mailed or e-mailed. The participant will then either post back the consent form in an addressed envelope, or sign and scan the consent form back to the study team.

To ensure informed consent has been obtained, the clinician will screen for any patient vulnerabilities before the initial phone call. This might include known difficulties with language or cognition. The clinician will call patients and carers and ask if they consent to be contacted by a research coordinator from Francis Health. This coordinator will be responsible for sending out and receiving the PICF. By way of this being managed by an independent party, this reduces the risk of patients feeling obliged to participate, and more comfortable to decline.

The PICF will include an outline of the sorts of questions the participant will be asked during the course of their interview, and participation in any co-design workshops. The limits of protecting their identity from other study participants will be explained as follows:

- Their identity can be protected if they participate in the interview only, with all identifying data being removed from transcripts and resultant reports or publications;
- Their identity can only be partially protected if they choose to participate in the Co-Design workshop, as they will become known to the wider study group. Their identity will not however be shared beyond this group, and the same caveats as explained above will apply to their interview.

In addition, prior to each interview commencing, the interviewer (Health Psychologist) will review the PICF, ask if the participant has any questions, and confirm the participants consent. All participants

For peer review only

will be offered the opportunity for a support person(s)/whanau to be present during the interview and co-design workshop.

For any participants who experience undue distress as a result of participation in the study, they will be offered supportive counselling via the Cancer Society (patients and carers), or an EAP service (staff members). All participants will be given the contact details of the national helpline 1737.

Qualitative assessment

Interviews with patients and whanau will focus on their experiences with CAR-T therapy. Interview questions will follow the themes of:

- Experiences through treatment;
- Parts that worked well; and
- Parts that could be improved on

"Experiences" will include:

- Practical elements, such as access to treatment, healthcare system navigation, transport, accommodation and financial impact.
- Informational needs (e.g. was enough information given at the right time, in ways you could understand).
- Emotional experiences - what was felt emotionally throughout the journey. (e.g. What was the emotional impact of treatment? Were your expectations met? Is there anything that could help make this process easier for you?)
- Cultural elements: were cultural and/or religious protocols respected and enacted throughout the process. Were there barriers along the way? What could be improved?

Interviews with clinicians and administrators will focus on their experiences with CAR-T therapy, following the themes of:

- System flow:
    - How well the system they work within facilitates access to CAR-T therapy – subjective and objective appraisals;
    - Their perceptions of the efficiency of system flow, as patients move through the CAR-T treatment pathway;
    - Barriers and enablers

- Staff experience of the CAR-T pathway:
    - Practical needs – is there anything that staff need that could improve the treatment pathway;
    - Informational needs – do all staff get the information they need at the right points along the journey;
    - Emotional needs – what is their emotional experience of CAR-T therapy. What is needed in terms of support resources for staff.

The interviews will be audio-recorded and transcribed. Resultant transcripts will be themed following a framework provided by Braun and Clarke (2006). Results of the interviews will be used to design an optimal care pathway which will be produced visually.

All participants will then be invited to participate in an online workshop, to review the optimal pathway. They will be asked to give their feedback on the design, and iterate any solutions that have been offered. The workshop will be facilitated by a Health Psychologist, and include staff members from the Malaghan Institute as well as consenting patients and whānau. All participants will be offered the opportunity to have a support person with them. This workshop, or focus group, will have specific ground rules designed to ensure a safe environment for all participants.

This project will employ co-design methodology to develop a patient- and whanau-centred pathway of care. Co-Design is a quality improvement framework that prioritises partnerships with service users to receive feedback and redesign services to meet users' needs. Following the methodology of Boyd et al (2012), this project will follow the six main elements of:

1. Engage: Recruiting key stakeholders, including clinical staff from Malaghan, and patients and whānau experienced with CAR T-cell therapy
2. Plan: interviewing staff to map out the necessary steps in the treatment pathway ('standard care'), pain points, and areas for improvement
3. Develop: Identifying key elements to include on the pathway to optimise patient- and whānau-centred care.
4. Decide: Assembling staff, patients and whānau together (via an online platform), to decide on what improvements to implement from the key elements

For peer review only

5. Change: Map out the requirements for making these improvements.

6. Data Storage

Patient information and consent forms will be stored in a secure area of Francis Health e.g. locked filing cabinet. The study specific number for each participant will be written on these forms, providing a link between the study number and identifiers. Consent forms will be destroyed after five years.

All electronic material will be stored on password protected computers, either within the Francis Health team or in the custody of the primary investigatory. This will include recordings of interviews, transcripts of the interviews, and recordings/summaries of the focus group session. The interviews will be stored under a unique anonymised study identifier, to prevent the participant being identified in the event of a data breach.

7. Study termination

Timing of study termination

Recruitment of participants shall cease at the earliest of the following:

- Recruitment of six patients; four support persons; five clinicians; and four administrators or support staff;
- Or June 2022

Publication and presentation of results

We anticipate that the study results will be published in an international scientific and medical journal. No participant will be identifiable from any published or presented outcomes of this study.

In addition, there will be a final report produced to Janssen Pharmaceuticals, the study sponsor, regarding optimal pathway design, requirements to enable optimal pathway implementation and delivery, and recommended next steps.

For peer review only

Destruction of study data

Study data will be kept by either Francis Health or Robert Fyfe, and then safely destroyed in a confidential manner.

References

Weinkove R et al (2021). Chimeric antigen receptor T-cells in New Zealand: challenges and opportunities. NZMA. 134 (1542): 97-108.

Boddy, C. R. (2016). Sample size for qualitative research. *Qualitative Market Research: An International Journal*, *19*(4), 426–432. https://doi.org/10.1108/QMR-06-2016-0053

Boyd, H., Mckernon, S., Mullin, B., & Old, A. (2012). Improving healthcare through the use of co-design. *NZMJ*, *29*(125). http://journal.nzma.org.nz/journal/125-1357/xxxx/

Braun, V., & Clarke, V. (2006). Using thematic analysis in psychology. *Qualitative Research in Psychology*, *3*(2), 77–101. https://doi.org/10.1191/1478088706qp063oa

Sandelowski, M. (1995). Sample size in qualitative research. *Research in Nursing & Health*, *18*(2), 179–183. https://doi.org/10.1002/NUR.4770180211

For peer review only

SRQR Guidelines, assessed for RE-TELL study (Fyfe R, et al).

Table 1

Standards for Reporting Qualitative Research (SRQR)[a]

No.	Topic	Item
	**Title and abstract**	
S1	Title	Concise description of the nature and topic of the study Identifying the study as qualitative or indicating the approach (e.g., ethnography, grounded theory) or data collection methods (e.g., interview, focus group) is recommended
S2	Abstract	Summary of key elements of the study using the abstract format of the intended publication; typically includes background, purpose, methods, results, and conclusions
	**Introduction**	
S3	Problem formulation	Description and significance of the problem/phenomenon studied; review of relevant theory and empirical work; problem statement
S4	Purpose or research question	Purpose of the study and specific objectives or questions
	**Methods**	
S5	Qualitative approach and research paradigm	Qualitative approach (e.g., ethnography, grounded theory, case study, phenomenology, narrative research) and guiding theory if appropriate; identifying the research paradigm (e.g., postpositivist, constructivist/ interpretivist) is also recommended; rationale[b]
S6	Researcher characteristics and reflexivity	Researchers' characteristics that may influence the research, including personal attributes, qualifications/experience, relationship with participants, assumptions, and/or presuppositions; potential or actual interaction between researchers' characteristics and the research questions, approach, methods, results, and/or transferability
S7	Context	Setting/site and salient contextual factors; rationale[b]
S8	Sampling strategy	How and why research participants, documents, or events were selected; criteria for deciding when no further sampling was necessary (e.g., sampling saturation); rationale[b]
S9	Ethical issues pertaining to human subjects	Documentation of approval by an appropriate ethics review board and participant consent, or explanation for lack thereof; other confidentiality and data security issues
S10	Data collection methods	Types of data collected; details of data collection procedures including (as appropriate) start and stop dates of data collection and analysis, iterative process, triangulation of sources/methods, and modification of procedures in response to evolving study findings; rationale[b]
S11	Data collection instruments and technologies	Description of instruments (e.g., interview guides, questionnaires) and devices (e.g., audio recorders) used for data collection; if/how the instrument(s) changed over the course of the study
S12	Units of study	Number and relevant characteristics of participants, documents, or events included in the study; level of participation (could be reported in results)
S13	Data processing	Methods for processing data prior to and during analysis, including transcription, data entry, data management and security, verification of data integrity, data coding, and anonymization/deidentification of excerpts
S14	Data analysis	Process by which inferences, themes, etc., were identified and developed, including the researchers involved in data analysis; usually references a specific paradigm or approach; rationale [b]
S15	Techniques to enhance trustworthiness	Techniques to enhance trustworthiness and credibility of data analysis (e.g., member checking, audit trail, triangulation); rationale[b]
	**Results/findings**	
S16	Synthesis and interpretation	Main findings (e.g., interpretations, inferences, and themes); might include development of a theory or model, or integration with prior research or theory
S17	Links to empirical data	Evidence (e.g., quotes, field notes, text excerpts, photographs) to substantiate analytic findings
	**Discussion**	
S18	Integration with prior work, implications, transferability, and contribution(s) to the field	Short summary of main findings; explanation of how findings and conclusions connect to, support, elaborate on, or challenge conclusions of earlier scholarship; discussion of scope of application/ generalizability; identification of unique contribution(s) to scholarship in a discipline or field
S19	Limitations	Trustworthiness and limitations of findings

(Table continues)

(Continued)

No.	Topic	Item
	**Other**	
S20	Conflicts of interest	Potential sources of influence or perceived influence on study conduct and conclusions; how these were managed
S21	Funding	Sources of funding and other support; role of funders in data collection, interpretation, and reporting

[a]The authors created the SRQR by searching the literature to identify guidelines, reporting standards, and critical appraisal criteria for qualitative research; reviewing the reference lists of retrieved sources; and contacting experts to gain feedback. The SRQR aims to improve the transparency of all aspects of qualitative research by providing clear standards for reporting qualitative research.

[b]The rationale should briefly discuss the justification for choosing that theory, approach, method, or technique rather than other options available, the assumptions and limitations implicit in those choices, and how those choices influence study conclusions and transferability. As appropriate, the rationale for several items might be discussed together.

S1: Completed – page 1
S2: Completed – page 3
S3: Completed – page 6
S4: Completed – page 7
S5: Completed – page 8
S6: Completed – pages 9-10
S7: Completed – page 7
S8: Completed – page 7
S9: Completed – page 10
S10: Completed – pages 9-10
S11: Completed – page 10; supplemental file 1
S12: Completed – pages 11-12
S13: Completed – page 10; supplemental file 3
S14: Completed – page 10
S15: Completed – page 8
S16: Completed – pages 11-23
S17: Completed – pages 21-22
S18: Completed – pages 24-26
S19: Completed – page 28
S20: Completed – page 30
S21: Completed – page 30

For peer review only
